# Unveiling Tissue‐Specific RNA Landscapes in Mouse Organs During Fasting and Feeding Using Nanopore Direct RNA Sequencing

**DOI:** 10.1002/advs.202408054

**Published:** 2024-12-16

**Authors:** Chengfei Jiang, Ping Li, Haiming Cao

**Affiliations:** ^1^ Cardiovascular Branch, National Heart, Lung and Blood Institute National Institutes of Health Bethesda MD 20892 USA

**Keywords:** ATAC‐Seq, energy metabolism, nanopore direct RNA sequencing, poly(A) tail length, RNA m6A modification

## Abstract

Understanding tissue‐specific RNA landscapes is essential for uncovering the functional mechanisms of key organs in mammals. However, current knowledge remains limited, as short‐read RNA sequencing—the predominant method for assessing gene expression—depends on incomplete gene annotations and struggles to resolve the diverse transcripts produced by genes. To address these limitations, an integrative approach combining nanopore direct RNA sequencing (DRS), ATAC‐Seq, and short‐read RNA‐seq is used. This method enabled the analysis of RNA landscapes across major mouse organs under fasting and fed conditions, representing two extremes of the caloric cycle. This study uncovered tens of thousands of novel transcripts and identified hundreds of genes with tissue‐specific expression, revealing additional layers of regulated pathways within each organ that conventional short‐read RNA‐seq cannot resolve. By profiling transcript expression across multiple organs under identical conditions, it is conducted comparative analyses exposing significant differences in transcript isoforms and regulations. Moreover, nanopore DRS revealed dynamic changes in poly(A) tail length and m6A modifications of transcripts, many regulated in a tissue‐specific manner. These changes likely contribute to functional differentiation and metabolic specialization of various organs. Collectively, this findings reveal previously unrecognized layers of gene regulation, offering new insights into the metabolic basis of organ function.

## Graphical abstract

An integrative approach combining nanopore direct RNA sequencing (DRS), ATAC‐Seq, and short‐read RNA‐seq is utilized to analyze RNA landscapes across major mouse organs. This study uncovers tens of thousands of novel transcripts and genes as well as dynamic changes in poly(A) tail length and m6A modifications of transcripts, which can contribute to functional differentiation and metabolic specialization of various organs.

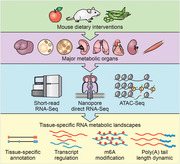

## 1 Introduction

Metabolism is the most fundamental requirement for life. Within a cell, metabolic pathways provide the energy needed for essential biochemical activities and the building blocks for organelles. Metabolites also play crucial roles as regulators of cellular signal transduction and gene expression.^[^ ^1^ ^]^ In an organism, particularly in mammals, key organs constantly engage in a dialogue to maintain systemic energy homeostasis.^[^ ^2^ ^]^ This metabolic interplay is essential for the proper function of each organ. Given that the metabolic status of all organs profoundly impacts their function, it is crucial to understand each organ's metabolic landscape under critical nutrient conditions, such as fasting and feeding. The predominant approach to assess the metabolic status of an organ is to examine its global gene expression, which is routinely carried out by short‐read RNA‐seq.^[^ ^3^ ^]^ This method, however, is constrained by multiple limitations. First, the analysis of short‐read RNA‐seq data usually relies on gene annotations which are currently incomplete.^[^ ^4^ ^]^ It is well established that one gene produces at least 10 transcript isoforms on average,^[^ ^5^ ^]^ many of which are conditionally expressed. As samples used to establish gene annotations are often confined to a few specific conditions, many conditionally expressed transcripts have not been annotated and cannot be analyzed by short‐read RNA‐seq. For example, we recently showed that over 50% of human liver transcripts identified by nanopore DRS are not included in current annotations (GENCODE).^[^ ^6^ ^]^ This incomplete annotation complicates the reliable assessment of gene expression. Second, short‐read RNA‐seq can only provide gene‐level information. The overlapping nature of short reads makes it challenging to resolve all transcripts, often resulting in the aggregation of reads associated with a gene to produce a single gene‐level expression value. This approach can obscure significant functional consequences of changes in specific transcripts. Finally, RNA modifications, such as m6A and poly(A) tails, contribute to RNA stability and protein translation, but are difficult to evaluate using conventional RNA‐seq. These limitations hinder the establishment of a comprehensive view of RNA metabolic landscapes.

To address these challenges, we implemented an integrative approach that combines nanopore direct RNA sequencing (DRS), ATAC‐Seq, and short‐read RNA‐seq. This comprehensive method allowed us to capture a more detailed and dynamic picture of RNA metabolic landscapes in key mouse organs under varying nutritional states. By integrating data from these three technologies, we were able to uncover novel transcripts and genes, as well as identify intricate regulatory mechanisms within each organ. More significantly, by profiling transcript expression in multiple organs under identical conditions, we also performed comparative analyses and identified substantial differences in transcript isoforms and regulations across all organs. Our findings shed light on the complex interplay of RNA modifications and gene expression, providing new insights into how metabolic states influence organ function.

## 2 Results

### 2.1 Tissue‐Specific RNA Metabolic Landscapes Revealed by Nanopore DRS

**Figure 1:**
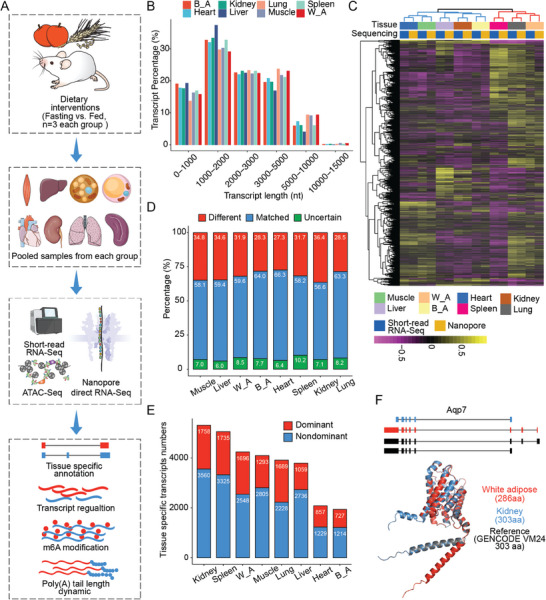
Tissue‐specific RNA metabolic landscapes revealed by Nanopore direct RNA sequencing (DRS). A) Schematic diagram of the experimental setup. Male C57BL/6 mice (n = 3 per group) were given either unrestricted access to water and a standard chow diet (fed) or subjected to a 24 h fasting period (fasting). Tissues including muscle, liver, heart, lungs, both white and brown adipose tissues, spleen, and kidney from the same treatment group (fed or fasting) were pooled uniformly. Each pooled sample was analyzed using short‐read RNA‐Seq, ATAC‐Seq, and nanopore DRS to assess tissue‐specific annotations, transcript‐level regulation, m6A modification, and dynamics of Poly(A) tail length in response to metabolic challenges. B) Bar plot displaying the distribution of transcript lengths across the tissues as measured by DRS. Each bar represents the frequency of transcripts within specified length ranges for different tissue types. C) Heatmap showing the pathway enrichment scores (GO term, Biological Process) analyzed by Gene Set Variation Analysis (GSVA) using gene‐level expression data from different fed tissue samples, derived from both short‐read RNA‐Seq and DRS. Each cell in the heatmap represents the enrichment score of a specific pathway for a particular tissue type. D) Comparison of DRS and reference annotations (GENCODE vM24) in different tissues. “Matched” transcripts are those with completely and exactly matched splicing sites compared to the reference annotation. “Different” transcripts are identified by differing isoform structures compared to those catalogued in the reference annotations. “Others” include isoforms with mismatched splicing sites or no actual overlaps with entries in the reference annotation. E) Bar plot displaying the number of dominant and nondominant expressed transcripts among tissue‐specific transcripts. Dominant transcripts are defined as the most highly expressed transcript within each gene. Each bar represents the count of these transcripts across different tissues. F) Top: Schematic diagram showing the structure of the dominant transcript of Aqp7 in the kidney (blue) and white adipose tissue (red), compared to the reference annotation (black). Bottom: Protein sequences of the dominant transcript of Aqp7 in the kidney (blue) and white adipose tissue (red) were predicted using CPC2. Additionally, their 3D structures were predicted using AlphaFold2, and the alignment of these structures is displayed to highlight structural similarities and differences.

To define tissue‐specific RNA metabolic landscapes across multiple organs in mice, we performed nanopore direct RNA sequencing (DRS) alongside short‐read RNA‐Seq and ATAC‐Seq analyses of eight mouse organs under fasting and fed conditions (**Figure** **1**A). Fasting and feeding are the two extremes of the caloric cycle and affecting the levels of a wide array of metabolites and hormones many of which impact the function of essential organs.^[^ ^7^ ^]^ DRS has been demonstrated to be able to distinguish between similar transcript subtypes, detect novel genes and transcripts, and quantify differential gene expression at the single transcript level with higher fidelity and accuracy than traditional short‐read RNA sequencing.^[^ ^8^ ^]^ We obtained a total of 18.12 million DRS long reads (78 x depth) and 1026.94 million paired‐end short‐read RNA‐Seq reads (416 x depth). The distribution of RNA lengths in DRS is similar across different tissues, predominantly falling within the 1000 to 2000 nucleotide range, with the liver showing the highest proportion in this range (Figure 1B; Figure S1A, Supporting Information). Analyzing the enrichment of biological pathways using gene expression data for each sample shows that short‐read RNA‐Seq and DRS data from various tissues cluster closely together, indicating their consistent ability to reveal biological functions (Figure 1C). Similar results were observed when comparing gene and transcript expression data between these two sequencing methods, further demonstrating the strong comparability between DRS and short‐read RNA‐Seq (Figure S1B, Supporting Information). To enhance the accuracy of DRS isoform annotations, we integrated short‐read RNA‐Seq sequence data to improve splicing site precision and used ATAC‐Seq chromatin accessibility information to correct RNA start sites. This integration was implemented using the FLAIR pipeline to develop tissue‐specific *de novo* gene annotations. Our results indicate that nearly one‐third of the isoforms in each tissue differ from those in the reference annotations, representing novel transcripts identified by DRS (Figure 1D and Table S1, Supporting Information). The relative abundance of novel transcripts in each tissue was also supported by short‐read RNA‐Seq, using DRS annotation as the reference (Figure S2A, Supporting Information). Among these novel transcripts, ≈85% of those from expressed protein‐coding genes in each tissue showed protein‐coding potential. This led to the identification of alternative coding sequences in ≈20%‐30% of the expressed proteins detected in DRS (Figure S2B–D, Supporting Information). To verify the existence of novel annotated proteins, we confirmed the presence of more than 60 novel proteins in mass spectrometry data from different mouse organs, including the heart, lung, liver, kidney, spleen, and muscle tissues (PXD041400) (Figure S3A,B, Supporting Information). One example is a novel transcript of the gene Ahnak (d0534183‐96c9‐43fe‐939e‐3c71b667415f), whose structure significantly diverges from the reference annotation, producing a distinct protein of 2455 amino acids (Figure S3C, Supporting Information). In the mass spectrometry data, this protein was identified with a unique peptide count of 75, sequence coverage of 67%, and a false discovery rate (FDR) Q‐value of less than 10^‐6 (Figure S3D, Supporting Information).

Furthermore, a systematic analysis of these tissue‐specific transcripts revealed that nearly one‐third are predominantly expressed compared to other isoforms within their respective genes, meaning they are the most highly expressed transcript in each gene (Figure 1E and Table S2, Supporting Information). Pathway analysis of these tissue‐specific transcripts revealed their involvement in important biological functions. For example, transcripts uniquely expressed in the kidney, liver, white adipose, and brown adipose tissues are enriched in the fatty acid metabolism pathway (Figure S4, Supporting Information). One notable example is the kidney‐ and white adipose‐specific isoforms of the gene Aquaporin 7 (Aqp7), which encodes aquaporins that facilitate the transport of water and small solutes, particularly glycerol, across cell membranes.^[^ ^9^ ^]^ The isoform structures of Aqp7 transcripts in these tissues differ from those in reference annotations. The kidney‐specific isoform, as well as the reference annotation (GENCODE vM24), encodes a protein of 303 amino acids, whereas the white adipose‐specific isoform encodes a protein of 286 amino acids. These differences in isoform structure result in substantial variations in the 3D conformation of the proteins (Figure 1F).

**Figure 2:**
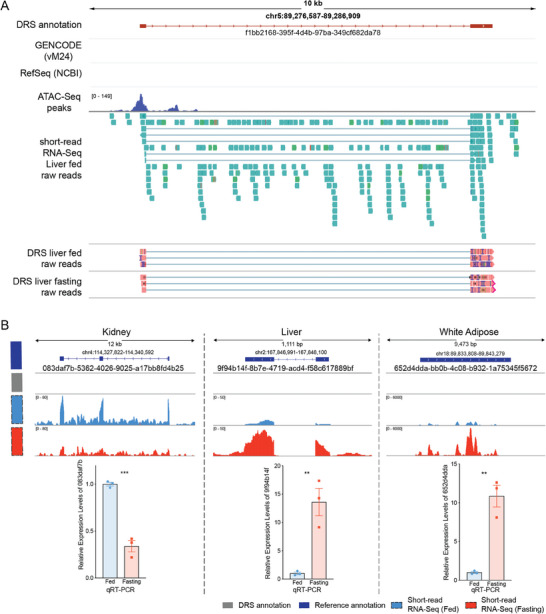
Novel genes discovered by DRS in different mouse tissues. A) Isoform schematics comparing the novel gene identified by DRS (f1bb2168‐395f‐4d4b‐97ba‐349cf682da78, shown in red) with reference annotations from GENCODE vM24 and RefSeq, where no transcript is present. The ATAC‐Seq read peaks from the fed mouse liver in the novel gene's genomic region are shown in blue. Raw reads from short‐read liver RNA‐seq (fed condition) are displayed in dark green, while the raw Nanopore DRS reads for both fed and fasting mouse livers are shown in light red. B) Top: Schematic representation of different novel transcripts identified in kidney, liver, and white adipose tissues by DRS. The novel isoforms identified are marked in dark blue, while their corresponding regions in the reference annotation are shown as lacking transcripts. Middle: short‐read RNA‐Seq read peaks for the fed group (blue) and the fasting group (red) are displayed. Bottom: Bar plot showing the relative expression levels of novel transcripts in different tissues under fed and fasting conditions (n = 3 for each group). Data are presented as mean ± SEM, with statistical significance indicated by **p < 0.01, ***p < 0.001 (two‐tailed unpaired Student's t‐test).

In addition to novel transcripts, we used the GffCompare algorithm to identify a substantial number of novel genes located in intergenic regions that are not annotated in current reference databases, such as GENCODE vM24.^[^ ^10^ ^]^ For example, ≈200 novel genes were identified in the kidney (Table S3, Supporting Information). As shown in **Figure** **2**A, at the genomic location chr5:89276587‐89286909, no gene is annotated in either GENCODE or RefSeq (NCBI) databases. However, DRS identified a novel gene, f1bb2168‐395f‐4d4b‐97ba‐349cf682da78, in liver tissue, which was also supported by short‐read RNA‐Seq and ATAC‐Seq data. While some of these novel genes are expressed across multiple tissues, many show tissue‐specific expression (Figure S5A–E, Supporting Information).

Moreover, DRS results indicated that these novel genes could be regulated by fasting, which was further validated by both short‐read RNA‐Seq and qRT‐PCR in different tissues. For instance, DRS identified the novel transcript 083daf7b‐5362‐4026‐9025‐a17bb8fd4b25 in the kidney, which is not annotated in reference databases. DRS showed that this transcript was downregulated during fasting, a result corroborated by short‐read RNA‐Seq, which exhibited a consistent decrease in expression peaks in the fasting group. This downregulation was further confirmed by qRT‐PCR, revealing a significant reduction in kidney expression (Figure 2B). Similarly, qRT‐PCR verified that 9f94b14f‐8b7e‐4719‐acd4‐f58c617889bf was upregulated in the liver, and 652d4dda‐bb0b‐4c08‐b932‐1a75345f5672 was upregulated in white adipose tissue (Figure 2B). Therefore, our approach of simultaneously analyzing multiple tissues under identical conditions is critical for the comprehensive identification of these novel genes. These findings highlight the functional significance of tissue‐specific transcript isoforms.

### 2.2 Alternative Splicing Dynamics in Response to Metabolic Stimuli Across Tissues

**Figure 3:**
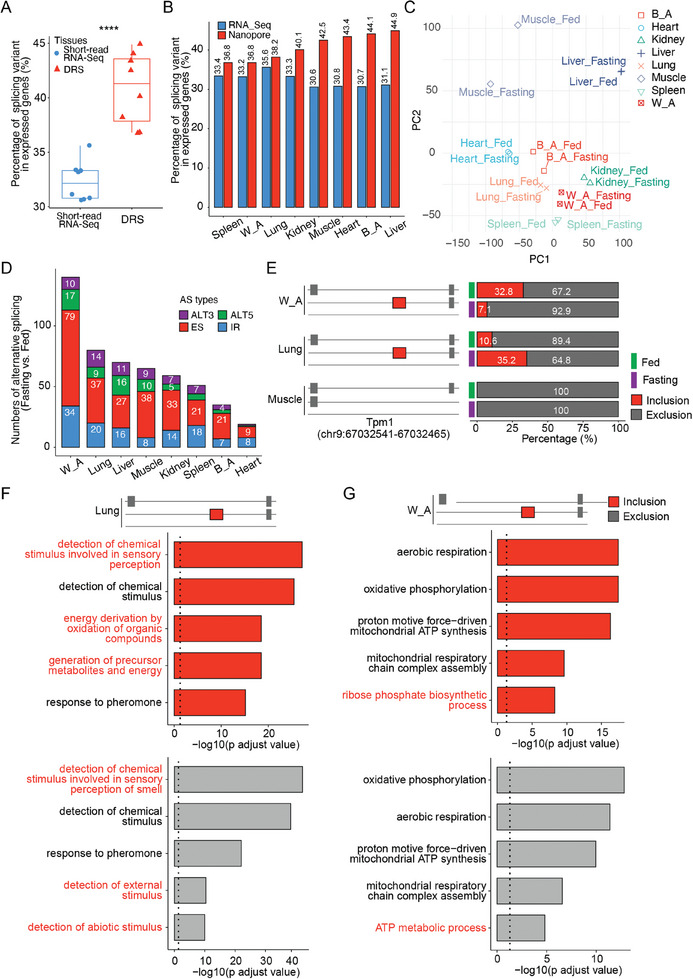
Alternative splicing (AS) dynamics in response to metabolic stimuli across tissues. A) Boxplots display the percentage of splicing variants in expressed genes across different tissues, analyzed separately using short‐read RNA‐Seq and nanopore DRS. The center line represents the median, while the boxes indicate the interquartile range. Genes were filtered based on expression in at least one condition (fed or fasting) for each tissue. Each data point corresponds to the percentage for an individual tissue. T‐tests were conducted to compare RNA‐Seq and DRS results, with statistically significant differences marked by asterisks (****p < 0.0001). B) Barplot shows the percentage of splicing variants in expressed genes from different tissues using RNA‐Seq and nanopore DRS, respectively. C) PCA plot of splicing events across samples subjected to different treatments. D) Bar plot displaying the numbers of significantly changed AS events between fasting and fed states in different tissues. AS events with a p‐value less than 0.01 were considered to be significantly altered, and different AS types are distinguished by different colors. E) Left: Schematic diagram of the Tpm1 exon skip event in the region (chr9:67032541‐67032465) from different tissues. The gray isoform represents exon exclusion, while the red isoform indicates exon inclusion. Right: Proportions of exon exclusion (gray) and inclusion events (red) in the specified region across different tissues in fed and fasting treatment groups. F,G) Top: Schematic diagram illustrating the Tpm1 exon skipping event in the genomic region (chr9:67032541‐67032465) across different tissues. Bottom: Barplot displaying the top 5 enriched pathways for transcripts associated with exon inclusion (red) and exon exclusion (gray) skipping events in lung (F) and white adipose tissues (G). The transcript expression signatures and correlation analysis were conducted using short‐read RNA‐Seq data downloaded from GSE132040 (n = 55 per tissue). Genes that significantly correlated with the exon skipping events (Person's correlation p‐value < 0.01 and |R| > 0.3) were subjected to GO Term pathway analysis. The top 5 enriched pathways, ranked by adjusted p‐value, are shown.

Alternative splicing (AS) is one of the key drivers of transcript diversity of a gene.^[^ ^11^, ^12^ ^]^ However, conventional RNA‐seq is limited in its ability to reliably define AS events due to the short length of its reads, a challenge that can be fully addressed by nanopore DRS. Indeed, DRS detected a significantly higher percentage of alternative splicing compared to short‐read RNA‐seq across all tissues (**Figure** **3**A,B). PCA analysis of alternative splicing events based on nanopore DRS demonstrates that transcripts from the same tissues cluster closely, whereas those from different tissues show significant differences in distribution (Figure 3C). This highlights the pronounced tissue specificity associated with the occurrence of alternative splicing. Using the FLAIR pipeline, we characterized four significantly altered alternative splicing (AS) events—Intron Retention (IR), Alternative 3′ Splicing (ALT3), Alternative 5′ Splicing (ALT5), and Exon Skipping (ES)—between fasting and fed conditions, based on the default settings. During the fasting treatment, compared to the fed group, white adipose tissue exhibited the most significant changes in alternative splicing events. Additionally, exon skipping types of alternative splicing events predominate across all tissue groups (Figure 3D and Table S4, Supporting Information). Moreover, the regulation of alternative splicing under fasting conditions also varies among different tissues. Pathway analysis of these significantly altered splicing events revealed notable functional differences across tissues in response to fasting treatment (Figure S6A, Supporting Information). For instance, tropomyosin 1 (Tpm1), which is implicated in stabilizing cytoskeleton actin filaments and regulating various important intracellular functions, is one such example.^[^ ^13^ ^]^ Studies have shown that the diversity of its functions depends on different isoforms.^[^ ^14^ ^]^ In our study, we found that in white adipose tissue under fasting conditions, there was a significant increase in exon skipping (ES) events in the region of chr9:67032541‐67032465. In contrast, in lung tissue, the ES events in this region significantly decreased, and in muscle tissue, there were no significant changes. These changes lead to diversity in Tpm1 isoforms during fasting and fed treatments (Figure 3E). The regulatory networks of these alternative splicing‐related transcripts differ significantly between lung and white adipose tissues. In the lung, the associated pathways are mostly related to the detection of chemical stimuli, whereas in white adipose tissue, they are primarily related to ATP synthesis pathways (Figure 3F,G). Additionally, we employed the SUPPA2 pipeline to characterize additional splicing types, including alternative first exons (AF), alternative last exons (AL), and mutually exclusive exons (MX). These analyses revealed that alternative splicing regulation under fasting conditions varies across tissues, with AF events predominating in all tissue groups (Figure S6B–E, Supporting Information). Collectively, these results underscore the tissue‐specific regulation of alternative splicing and its crucial role in generating isoform diversity across different conditions.

### 2.3 Transcript‐Level RNA Landscapes Revealed by Nanopore DRS Complement Gene‐Level Information Across Tissues

In this work, we used three sequencing technologies each of which revealed important aspects of tissue‐specific RNA metabolic landscapes in key mouse organs. For example, short‐read RNA‐Seq revealed that tissues demonstrated significant responses under fasting conditions, and that gene expression trends and responses to fasting varied significantly across different tissues (Figure S7A and Table S5, Supporting Information). For instance, the liver exhibited a significant activation of the fatty acid degradation pathway and an inhibition of steroid biosynthesis during fasting, aligning with current scientific understanding (Figure S7B, Supporting Information). The ATAC‐Seq results showed differences in chromatin accessibility across various tissues. For example, the lung and kidney had the highest overall promoter region accessibility among all tissues, while the liver and spleen had relatively lower levels of accessibility. In response to fasting, the liver showed an increase in promoter region accessibility, whereas both brown and white adipose tissues exhibited a decreasing trend (Figure S7C, Supporting Information). Interestingly, changes in chromatin accessibility at transcription factor binding sites in response to fasting varied substantially across different tissues. For instance, Nuclear Respiratory Factor 1 (Nrf1), a crucial regulator of cholesterol homeostasis,^[^ ^15^ ^]^ showed a significant increase in enrichment in the liver during fasting, while its enrichment decreased in brown adipose tissue (Figure S7D and Table S6, Supporting Information). Our results indicate that most tissues, excluding the heart and spleen, exhibited ≈20%‐30% of differentially expressed genes (DEGs) with notable changes in promoter region accessibility (Figure S8A, Supporting Information). Notably, in the majority of tissues—such as the liver, muscle, lung, brown adipose tissue, and kidney—we observed a significant positive correlation between gene expression changes and promoter region accessibility fold changes during fasting, suggesting a positive association between promoter accessibility and the expression levels of corresponding genes (Figure S8C–G, Supporting Information). In contrast, white adipose tissue showed a negative association between gene expression changes and promoter accessibility dynamics (Figure S8B, Supporting Information), while the heart and spleen exhibited no clear correlations (Figure S8H,I, Supporting Information).

**Figure 4:**
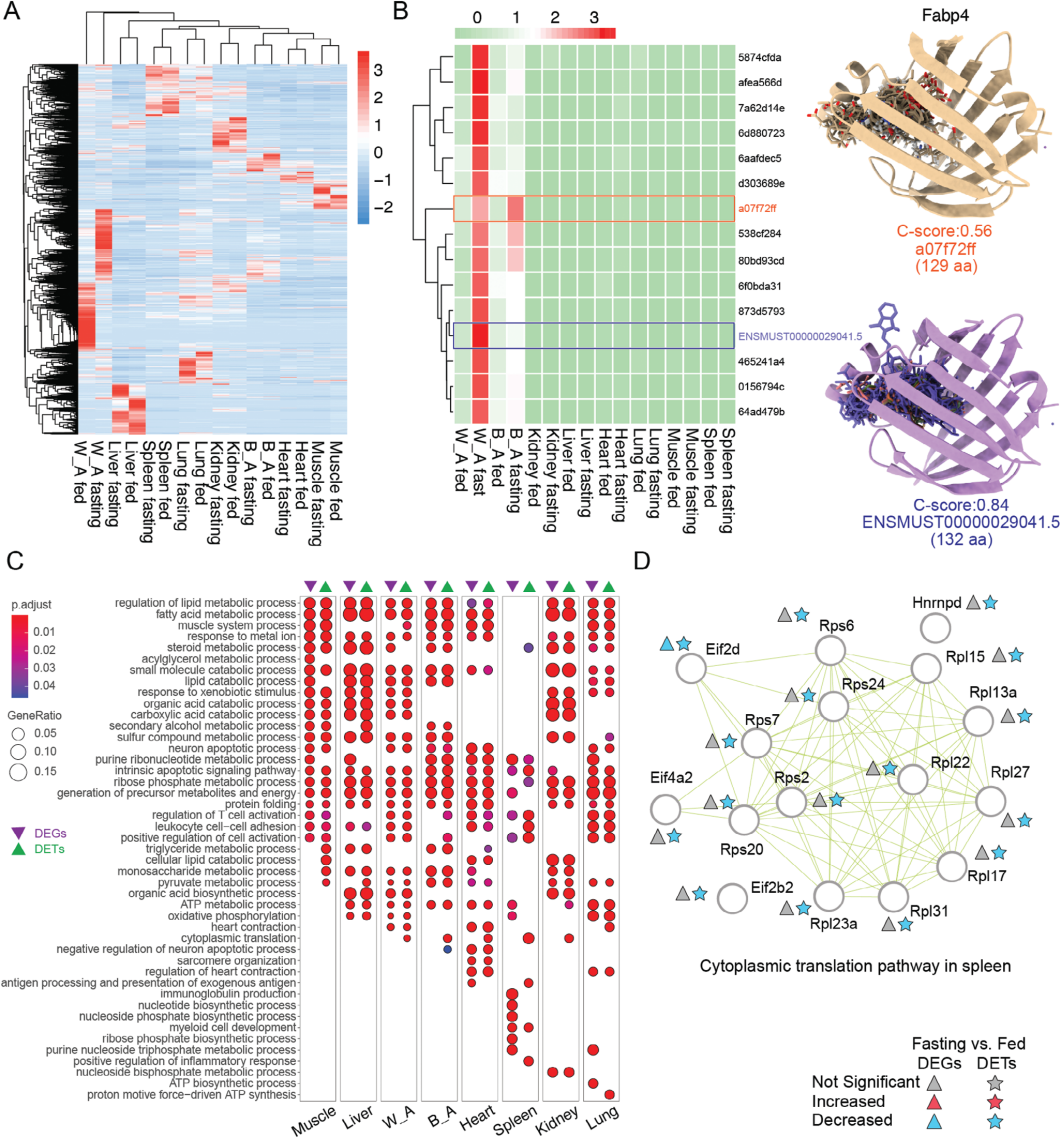
Transcript‐level RNA landscapes revealed by nanopore DRS complement gene‐level information across tissues. A) Heatmap of transcript‐level expression in each sample. B) Left: Heatmap showing the transcript expression levels of Fabp4 in different tissues. The dominant transcripts in white adipose tissue and brown adipose tissue are highlighted. Right: The 3D structure and fatty acid binding capacities of dominant transcripts from white adipose tissue (purple) and brown adipose tissue (brown), predicted by I‐TASSER. C) Comparisons of the top enriched pathways (GO term, Biological Process) for differentially expressed genes (DEGs, purple) and differentially expressed transcripts (DETs, green) in different tissues during fasting treatments from DRS. D) Gene network illustrating changes in gene‐level and transcript‐level expression within the triglyceride metabolic process pathway in muscle tissue during fasting treatment. In the network, DEGs are marked with triangles, and DETs with pentagons. All the DEGs and DETs were from the DRS. Upregulated entities are indicated in red, downregulated entities in blue, and those without significant changes are marked in gray.

As our understanding of gene regulation deepens, the significance of differential regulation among transcripts within a gene in controlling gene function is increasingly acknowledged. Currently, gene and transcript regulation largely rely on RNA‐seq, yet the short‐read nature of RNA‐seq provides good resolution at the gene level but imposes considerable constraints at the transcript level.^[^ ^16^ ^]^ To gain further insights into RNA transcript regulations, we employed DRS to directly capture changes in full‐length RNA transcripts. Moreover, unlike previous nanopore DRS studies that often focused on a single organ, we simultaneously analyzed multiple key mouse organs under two feeding conditions. This approach allowed us to compare not only tissue‐specific expressions but also the regulation mechanisms specific to each tissue. Our results indicate that transcript‐level expression demonstrates great tissue specificity (**Figure** **4**A). Furthermore, under fasting conditions, the changes in transcript expression exhibit significant differences across various tissues. For instance, in white adipose tissue, a significantly larger portion of transcripts exhibit distinct expression patterns in fed and fasting conditions, whereas these patterns are less pronounced in other tissues (Figure 4A). Fatty Acid Binding Protein 4 (Fabp4) is specifically expressed in adipose tissues, where it facilitates the binding of fatty acids, thereby regulating fatty acid uptake, transport, and metabolism.^[^ ^17^ ^]^ Under fasting conditions, we noted significant enrichment of Fabp4 in both white and brown adipose tissues (Figure 3B and Table S7, Supporting Information). However, the transcripts predominantly expressed vary between these tissues: in white adipose tissue, transcript ENSMUST00000029041.5 (purple) encodes a protein of 132 amino acids, whereas in brown adipose, transcript a07f72ff (orange) encodes a protein of 129 amino acids. Substrate binding analysis showed notable differences in their fatty acid binding capacities (I‐TASSER c‐score 0.84 for ENSMUST00000029041.5, I‐TASSER c‐score 0.56 for a07f72ff), illustrating the existence of divergent transcript regulation across different tissues that may have specific regulatory roles (Figure 4B). Further analysis compared DEGs and differentially expressed transcripts (DETs) both identified through DRS under fasting conditions. We observed that although a substantial portion of genes did not exhibit significant changes at the gene level, significant variations were noted in their internal transcripts. Pathway analysis of biological functions revealed that both DEGs and DETs are enriched in some common pathways; however, distinct differences are also apparent. For instance, in spleen tissue, both DEGs and DETs are enriched in pathways related to the purine ribonucleotide metabolic process, but DETs also show enrichment in processes such as the cytoplasmic translation pathway (Figure 4C). In this pathway, only one gene, Eif2d, demonstrated consistent upregulation at both the DEG and DET levels. In contrast, other enriched genes, such as Rps6, Rpl15, and Hnrnpd, showed no significant changes at the gene level, but specific transcripts of these genes were significantly downregulated (Figure 4D). These findings underscore the importance of transcript‐level analyses as a complement to gene‐level changes, revealing comprehensive tissue‐specific RNA metabolic landscapes.

### 2.4 Polyadenylation Variabilities Across Different Tissues

**Figure 5:**
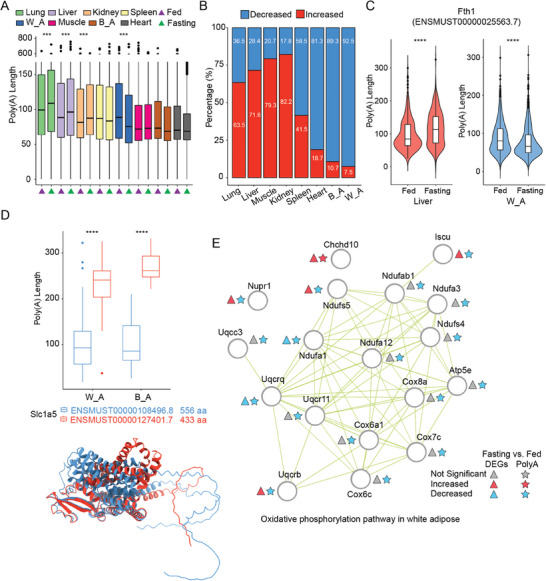
Polyadenylation variabilities across different tissues. A) Boxplot of Poly(A) tail length distribution in each sample. Different tissues are indicated by different colors, and fasting and fed treatments are marked with different colored triangles at the bottom. Significant differences are denoted with ***p<0.001, determined by a two‐tailed unpaired Student's t‐test comparing fasting and fed treatments in each tissue. B) Percentage of transcripts with significantly increased and decreased Poly(A) tail lengths in different tissues in response to fasting treatment. Significant shifts in Poly(A) tail lengths were determined using a cutoff of length changes greater than 10 nucleotides and a p‐value less than 0.05. C) Violin plot showing the Poly(A) tail length of the Fth1 transcript ENSMUST00000025563.7 in liver and white adipose tissue under fed and fasting treatments. ****p<0.0001, two‐tailed unpaired Student's t‐test. D) Top: Boxplot displaying the Poly(A) tail lengths of two different transcripts of Slc1a5 under fed conditions: ENSMUST00000108496.8 (blue) and ENSMUST00000127401.7 (red), in white and brown adipose tissues respectively. Bottom: 3D protein models of ENSMUST00000108496.8 (blue, 556 amino acids) and ENSMUST00000127401.7 (red, 433 amino acids) as predicted by AlphaFold2. E) Gene network of changes in gene‐level and Poly(A) tail length changes within the oxidative phosphorylation pathway in white adipose tissue during fasting treatment.

Emerging evidence underscores the vital roles of Poly(A) tail length in mRNA quality control, serving as a signal for both mRNA degradation and translation,^[^ ^18^ ^]^ but our systematic understanding of Poly(A) tail dynamics across different organs remains limited.^[^ ^19^, ^20^ ^]^ Leveraging poly(A)‐enriched Nanopore DRS technology, it is now feasible to detect the length of native RNA poly(A) tails.^[^ ^21^ ^]^ Poly(A) tail lengths exhibit distinct distribution patterns across various tissues. For instance, lungs have the longest average Poly(A) tail length, ≈100 nucleotides, whereas hearts have the shortest, ≈70 nucleotides (**Figure** **5**A and Table S8, Supporting Information). Additionally, during fasting treatment, about half of the tissues exhibited overall changes in Poly(A) tail length, with variations observed across different tissues. Relative to the fed condition, Poly(A) tail lengths increase in the lungs, liver, and kidneys under fasting, but decrease in white adipose tissue (Figure 5A). Further analysis of transcripts that undergo significant Poly(A) length changes shows that in lungs, liver, muscles, and kidneys, the majority of significantly altered transcripts increase, while in tissues like adipose, there is a predominant decrease (Figure 5B; Figure S9A and Table S9, Supporting Information). Research shows that the length of the Poly(A) tail correlates with translation efficiency, with longer Poly(A) tails leading to more efficient mRNA translation.^[^ ^22^ ^]^ Taking the liver as an example, under fasting conditions, transcripts with reduced Poly(A) tail lengths are enriched in the fatty acid metabolic process. Conversely, transcripts with increased lengths are enriched in pathways involved in the generation of precursor metabolites and energy (Figure S9B, Supporting Information). This may relate to the liver suppressing fat synthesis under fasting conditions to increase energy output. Interestingly, the changes in Poly(A) tail length of the same transcripts under fasting treatment can vary between different tissues. For example, the transcript ENSMUST00000025563.7 of Ferritin Heavy Chain 1 (Fth1), a key regulator of iron metabolism,^[^ ^23^ ^]^ showed a significant increase in Poly(A) tail length in the liver, while in white adipose tissue, it significantly decreased (Figure 5C). Additionally, different transcripts of the same gene exhibit variations in Poly(A) tail length. For example, one transcript of Solute Carrier Family 1 Member 5 (Slc1a5),^[^ ^24^ ^]^ ENSMUST00000108496.8 (blue, forming a 556 aa protein), has a significantly shorter Poly(A) tail length compared to another transcript, ENSMUST00000127401 (red, forming a 433 aa protein), in both white and brown adipose tissues. This suggests that differences in Poly(A) tail length among transcripts may be an important mechanism for regulating the functions of transcripts within a gene (Figure 5D). Similarly to the DETs results, transcripts with changes in Poly(A) tail length are involved in pathways that not only share functions with DEGs but also provide significant supplementary roles. For example, in white adipose tissue, transcripts with significant changes in Poly(A) tail length are notably enriched in the Oxidative phosphorylation pathway compared to DEGs (Figure S9C, Supporting Information). Moreover, in this pathway, most genes do not exhibit significant changes at the gene level but show a reduction in Poly(A) tail length, suggesting an important regulatory role of Poly(A) tail length dynamics in white adipose tissue within this pathway (Figure 5E). The above results reveal the dynamics of Poly(A) tail length in different tissues and among different transcripts within the same gene, supporting their essential role in metabolic regulation.

### 2.5 Metabolic Responsive Dynamics of RNA m6A Modification Across Tissues

**Figure 6:**
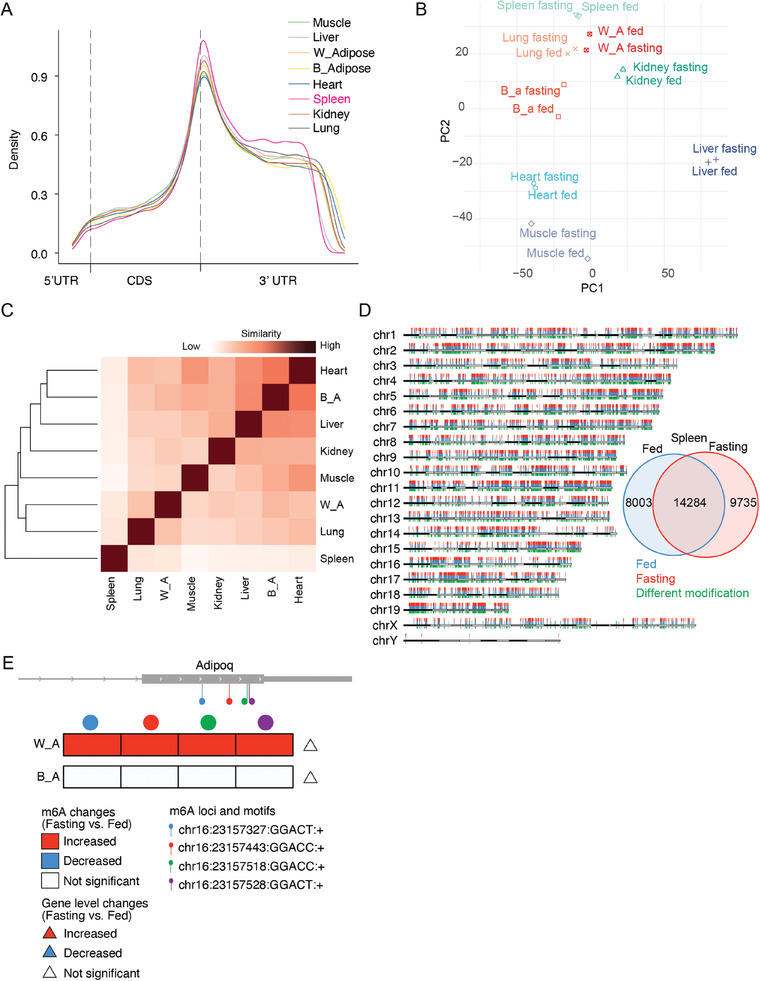
Metabolic responsive dynamics of RNA m6A modification across tissues. A) Metagene analysis showing the distribution of modifications along the gene body in different tissues. The MetaPlotR pipeline was used to evaluate the distribution of m6A modifications within the gene bodies of known protein coding genes based on the GENCODE vM24 reference annotation. B) PCA plot of m6A modification events across different tissues with different treatments. C) Heatmap displaying the similarities of m6A modifications across various tissues. Clustering was performed using a distance matrix derived from Z‐score normalized m6A frequency data. Each matrix element represents the pairwise distance between two samples, with distance inversely reflecting similarity—samples with lower distances share more similar m6A modification profiles. D) Top left: Distribution of m6A modification sites in spleen tissue for fed (blue) and fasting (red) conditions, with differences highlighted in green. Bottom right: Venn diagram showing the overlap of m6A modifications between the fed and fasting treatments. E) Top: A schematic diagram illustrating the loci of m6A modifications on the Adipoq gene body. Bottom: A heatmap displaying significant changes in m6A modifications during fasting treatment in the Adipoq gene body in white and brown adipose tissues. Different modification loci are represented as circles in various colors on the heatmap. The m6A modification changes are depicted within the heatmap, with DEGs indicated by triangles displayed on the right side of the heatmap. Increases are depicted in red, decreases in blue, and no significant changes in white.

Growing evidence supports that RNA N6‐methyladenosine (m6A) modifications play a pivotal role in regulating RNA processing, stability, and translation.^[^ ^25^, ^26^ ^]^ However, little is known about the systemic changes in m6A modifications across different tissues in response to metabolic challenges. Nanopore DRS has been shown to be highly effective in detecting native RNA modifications in biological samples, offering significant sensitivity and specificity.^[^ ^27^, ^28^, ^29^ ^]^ Utilizing this capability of DRS, we further analyzed the m6A modification patterns of transcripts across different tissues. M6A modifications in various tissues primarily occur in the CDS and 3′ UTR regions, with the modification locus distribution being similar across all tissues except for the spleen. In the spleen, m6A modifications in the 3′ UTR region are relatively farther from the 3′ end compared to other tissues (**Figure** **6**A). By comparing the frequency of m6A modifications across different tissues, we observed significant variations in m6A modification patterns among them. Some tissues, such as white adipose tissue and lungs, have relatively similar modification patterns, while others, like the spleen, show significant differences compared to other tissues (Figure S10A, Supporting Information). These findings are also supported by PCA analysis of m6A modification frequencies across tissues. The PCA analysis revealed that tissue samples cluster well according to tissue type, reflecting the tissue specificity of m6A modifications (Figure 6B and Table S10, Supporting Information). Furthermore, taking the spleen as an example, we found that m6A modifications are relatively evenly distributed across all chromosomes. Consistently, sample similarities of m6A modifications results showed that different tissues exhibited significant differences in m6A modification patterns. For instance, white adipose tissue and lungs displayed relatively similar modification patterns, while tissues like the spleen showed significant differences compared to others (Figure 6C). Additionally, the majority (>60%) of the modifications are common to both fed and fasting conditions, but there are also unique modification sites specific to each condition (Figure 6D). This suggests an important regulatory role for m6A modifications during fed and fasting states. Compared to the gene‐level changes in DEGs, m6A modifications provide another dimension of information about RNA changes. We further analyzed m6A modifications under fasting and fed conditions and compared them with the pathways enriched by DEGs. The results show that significantly changed m6A modifications share many pathways with DEGs, but also point to some unique pathways. For example, in muscle, both DEGs and significantly changed m6A modifications are remarkably enriched in muscle function‐related pathways, such as muscle system processes. Additionally, m6A modifications are also enriched in important functional pathways, such as protein folding and fatty acid metabolism (Figure S10B, Supporting Information). Meanwhile, changes in m6A modifications of genes in response to metabolism vary across different tissues (Table S11, Supporting Information). Taking Adiponectin, C1Q, and Collagen Domain Containing (Adipoq) as an example, which is involved in the control of fat metabolism and insulin sensitivity,^[^ ^30^ ^]^ multiple m6A modification sites exist in its region on chr16:23157327‐chr16:23 157 528, and these modifications significantly increase in white adipose tissue under fasting conditions, although there is no significant change at the gene level. In contrast, in brown adipose tissue, there is neither a change in gene expression level nor in m6A modifications (Figure 6E). These results demonstrate the divergence in m6A modifications across different tissues and their varying responses to metabolic challenges, suggesting that m6A modifications may play an important role in metabolic functions in different tissues.

## 3 Discussion

The primary focus of this study is to delineate the tissue‐specific RNA metabolic landscapes in response to fasting, utilizing the integrative approach of nanopore DRS combined with short‐read RNA‐Seq and ATAC‐Seq. Unlike previous nanopore DRS studies that often focused on a single organ, we simultaneously analyzed multiple key mouse organs under two feeding conditions. This approach allowed us to compare not only tissue‐specific expressions but also the regulatory mechanisms specific to each tissue.

In our study, short‐read RNA‐seq and ATAC‐Seq provided comprehensive insights into the gene‐level changes and transcriptional regulation across different tissues under identical metabolic conditions. These technologies allowed us to map the broad landscape of gene expression and chromatin accessibility, revealing essential pathways and regulatory mechanisms at play during fasting. However, despite their broad utility, short‐read RNA‐seq primarily captures gene‐level changes, which may not fully represent the complexity of RNA processing and regulation. Nanopore DRS we utilized in this work demonstrated several advantages over traditional RNA‐seq. First, nanopore DRS does not rely on existing annotations and allows us to discover all detectable transcripts, which we used to build novel annotations of the transcriptomes of key mouse organs. Second, nanopore DRS allows for the detection of full‐length transcripts, providing a deeper understanding of transcriptomic dynamics that traditional methods might miss. One of the standout findings from using DRS is the discovery of tissue‐specific transcripts, many of which are novel and not cataloged in existing genomic databases. Our further analyses suggest that many of these tissue‐specific transcripts result from unique patterns of alternative splicing that are specific to each tissue type. In terms of functional implications, changes at the transcript level, as revealed by DRS, offer additional insights that extend beyond gene‐level variations. For example, different transcripts of the Fatty Acid Binding Protein 4 (Fabp4) are differentially activated in various tissues, potentially playing distinct roles in metabolic processes specific to each tissue. In muscle tissue, while the majority of gene‐level expressions within the triglyceride metabolic process pathway show no significant change, the transcript‐level variations are profound and indicative of regulatory mechanisms at work that are not apparent at the gene level. Moreover, DRS has uncovered post‐transcriptional modifications that add another layer of regulation, such as the dynamics of Poly(A) tail length and m6A modifications. These findings illustrate significant tissue‐specific differences in Poly(A) tail lengths and their changes in response to fasting, highlighting the importance of transcript‐level regulation in metabolic responses. For example, the Poly(A) tail length of transcripts shows significant variability between different tissues and even between different transcripts of the same gene within a tissue, suggesting a finely tuned mechanism of mRNA stability and translation efficiency that responds to metabolic needs. Additionally, DRS has revealed that m6A RNA modifications also exhibit strong tissue specificity, underscoring the critical role of these modifications in tissue‐specific regulatory processes. The varied m6A modification patterns across tissues and their changes in response to fasting conditions suggest that m6A modifications play significant roles in the metabolic adaptation and regulatory landscape of different tissues. Overall, these results highlight that DRS provides a comprehensive atlas of RNA metabolic responses across various tissues, offering new insights into the complex regulatory networks at play during metabolic challenges. This level of detail enhances our understanding of the molecular mechanisms underlying tissue‐specific metabolic adaptations and offers new perspectives on the metabolic basis of organ function.

This study unveils RNA metabolic landscapes across various tissues during fasting but faces inherent limitations that necessitate further exploration. The metabolic challenge employed here is limited to fasting—a fundamental but singular type of metabolic stress.^[^ ^31^, ^32^ ^]^ Extending the research to include a variety of metabolic conditions and disease models would provide a broader, more comprehensive view of how RNA metabolism responds across tissues under different physiological stressors. The use of the C57BL/6 mouse strain, while beneficial due to its extensive characterization and common usage in research, introduces limitations due to genetic uniformity. Diverse genetic backgrounds in mice can influence the metabolic response to metabolic challenges and other stresses, suggesting that incorporating multiple strains could reveal a richer array of metabolic responses and regulatory mechanisms.^[^ ^33^, ^34^ ^]^ This approach would enhance the generalizability of the findings and help uncover genetic factors that may influence RNA metabolism. Additionally, the application of findings from mouse models to human studies presents some gaps, mainly due to physiological and metabolic differences between species.^[^ ^35^ ^]^ Furthermore, we used the GENCODE annotation as the reference database to identify novel transcripts and genes, as it integrates data from multiple sources to ensure accuracy and includes a broad range of verified gene annotations.^[^ ^36^, ^37^ ^]^ However, there is still the possibility that some of the novel transcripts we identified have been reported by other sources. Despite these differences, the insights gained from studying RNA metabolism in mouse organs under controlled experimental conditions provide a valuable framework. This framework can inform hypotheses and experimental designs in human metabolic research, although direct comparisons and translations must be approached with caution. We hope our work will not only advance the understanding of the metabolic basis of organ function in mouse models but also serve as a foundational reference for extending these studies to human metabolic research.

## 4 Experimental Section

### Animals

All procedures involving animals were conducted in strict accordance with the guidelines approved by the National Heart, Lung, and Blood Institute (NHLBI) Animal Care and Use Committee (approved animal protocol number H‐0248). Male C57BL/6 mice, sourced from the Jackson Laboratory, were acquired at 12 weeks of age. The mice were housed in groups of three per cage, with unrestricted access to water and a standard chow diet consisting of 24% kcal from protein, 14% kcal from fat, and 62% kcal from carbohydrates (NIH‐31, Harlan Teklad). Prior to the commencement of experiments, the animals were allowed a minimum acclimatization period of 10 to 14 days under these housing conditions. For the experimental treatments involving fasting and fed states (n = 3 per group), the mice were either provided with continuous access to food or subjected to a 24 h fasting period before euthanasia at 10 am for tissue collection. The tissues harvested included muscle, liver, heart, lungs, both white and brown adipose tissues, spleen, and kidney. These tissues were immediately preserved in liquid nitrogen post‐sacrifice to maintain their integrity for subsequent analyses.

### RNA Extraction

Tissue samples from the same experimental group were pooled equally and ≈15–40 mg of pooled frozen tissue powder was homogenized 20–25 times in a Dounce homogenizer with 1.2 ml of Trizol reagent (Invitrogen, Catalog No. 15 596 018) on ice. The homogenized samples were then transferred to new 2 ml nuclease‐free Eppendorf (EP) tubes and allowed to incubate on ice for 5 min. Following incubation, the lysates were centrifuged at 10 000 x g for 2 min at 4 °C. Subsequently, 1 ml of each supernatant was carefully transferred to new nuclease‐free 1.5 ml EP tubes. To each sample, 200 µl of chloroform per ml of supernatant was added, followed by gentle mixing. The mixtures were allowed to stand at room temperature for 5 min before being centrifuged at 12 000 x g for 10 min at 4 °C. Post‐centrifugation, 500 µl of the upper aqueous phase was transferred to new EP tubes. To this, 500 µl of isopropanol was added, and the samples were mixed gently and incubated at room temperature for 15 min. The samples were then centrifuged at 12 000 x g for 10 min at 4 °C, after which the supernatants were discarded. The RNA pellets were washed with 500 µl of 75% ethanol and centrifuged at 12 000 x g for 5 min at 4 °C. After discarding the supernatant, the washing step was repeated. The RNA pellets were either stored in 75% ethanol at −80 °C or processed further by removing the supernatants and air‐drying for 10 min. The dried RNA pellets were then resuspended in 100 µl of nuclease‐free water and used immediately for further analyses.

### RNA Purification, Short‐Read RNA‐Seq and Analysis

RNA samples extracted using the Trizol method were further purified with the MagMAX RNA extraction kit from Thermo Fisher Scientific (Catalog No. AM1830) to ensure high‐quality RNA for sequencing. For the construction of strand‐specific sequencing libraries, the Illumina TruSeq RNA Sample Prep Kit was employed. Subsequent paired‐end sequencing with a read length of 100 base pairs was conducted at the NHLBI DNA Sequencing and Genomics Core. Each sample was guaranteed a minimum yield of 100 million reads to ensure comprehensive coverage and depth of sequencing data.

The short‐read RNA‐Seq process was outlined in a previous study.^[^ ^35^ ^]^ Initially, raw FASTQ read files were processed for quality improvement using the fastp/0.23.2 tool, followed by quality checks performed with FastQC/0.11.8. Reads alignment was conducted using HISAT2/2.2.1.0 against the GRCm38 mouse genome, and the resulting alignments were quantified using featureCounts (subread/2.0) based on the mouse GENCODE vm24 annotation. Differential gene expression analysis was performed using Fisher's exact test on a gene‐by‐gene basis.^[^ ^38^, ^39^, ^40^ ^]^ The expression levels were normalized to counts per million (CPM). The fold change (FC) between fasting and fed conditions was calculated as (CPM _fasting_+0.01)/ (CPM _fed_+0.01). A threshold for significant differential expression was set at a log2(FC) greater than 0.5 and a p‐value less than 0.01 across the compared groups.

### RT‐qPCR Analysis

Fresh RNA was extracted and purified from liver, white adipose, and kidney tissues from three mice per group, as described. For reverse transcription, the SuperScript III First‐Strand Synthesis System (Invitrogen, Cat. 11 752 250) was used with 500 ng of RNA. Quantitative real‐time RT‐PCR was conducted using a ViiA 7 Real‐Time PCR System (Applied Biosystems Inc.), with Actb as the internal control to measure the expression levels of the novel genes. The PCR program consisted of an initial enzyme activation step of 2 min and 30 s at 95 °C, followed by 40 cycles of 15 s at 95 °C and 1 min at 60 °C. A melting curve analysis was performed to verify the specificity of the PCR products. The full primer sequences used were provided in Table S12 (Supporting Information).

### ATAC‐Seq Library Preparation, Sequencing and Analysis

Tissue powder from each organ of the same experimental group was pooled in equal proportions. The nuclei isolation, as previously described,^[^ ^6^ ^]^ involved processing ≈40 mg of pooled tissue powder per run. The tissue was homogenized, filtered, and centrifuged to separate supernatants. Iodixanol solutions (Sigma‐Aldrich, Cat. D1556) were used to create density gradients for further centrifugation. The top layer containing nuclei was isolated and the nuclei were resuspended. After another centrifugation step, the nuclear pellet was treated with a mixture containing transposase (Illumina, Cat. 20 034 210) and incubated. DNA was then purified using the MinElute Reaction Cleanup Kit (Qiagen, Cat. 28 206) and either stored at −80 °C or used for ATAC‐Seq library preparation. The generation of ATAC‐Seq DNA libraries was conducted following the Kaestner lab's pipeline.^[^ ^41^ ^]^ After purification, DNA samples were amplified using TruSeq i7 index primers (Illumina) through PCR. Subsequent purification and size selection of the amplified DNA were carried out using AMPure XP beads (Beckman Coulter, Cat. A63881), which helped remove primer dimers and any fragments exceeding 1000 bp. The quality and quantity of the library were assessed using a Bioanalyzer and Qubit respectively, ensuring optimal library preparation. Finally, the prepared library was sequenced at the NHLBI DNA Sequencing and Genomics Core.

The processing of raw FASTQ reads was carried out using the established pipeline from the Kundaje Laboratory, available at https://github.com/kundajelab/atac_dnase_pipelines. This pipeline involves several steps crucial for high‐quality data analysis, starting with adapter trimming to prepare the reads. Subsequently, the trimmed reads were aligned to the genome using Bowtie2, employing the same reference genome configuration as in the humanized mouse short‐read RNA‐Seq analysis. Post‐alignment, reads were further refined by removing unmapped reads and duplicates to ensure that only high‐quality, unique reads were used for downstream analysis. Peak calling was performed using MACS2, which identified regions of open chromatin based on the filtered reads. Significant chromatin open regions were defined by a false discovery rate (FDR) Q‐value of less than 0.01, highlighting areas of potential regulatory activity. The identified peaks of these open regions were annotated and visualized using the R package ChIPseeker/3.19.^[^ ^42^ ^]^

Transcription factor (TF) motif enrichment and activity analysis were carried out using HOMER/4.11.1, specifically the findMotifsGenome.pl function, with a motif length set to 8 nucleotides.^[^ ^43^ ^]^ The motifs identified as enriched were ranked according to their p‐values, and the top five motifs for each organ were selected for visualization on dot plots. To further analyze the TF regulatory activities in the identified open chromatin regions, the HINT‐BC/0.13.2 software was employed, utilizing its “footprinting” function.^[^ ^44^ ^]^ This tool helps in detecting TF binding sites based on the specific patterns of protection against nuclease activity, indicative of TF binding. Additionally, differential activity between two conditions was analyzed using the “differential” function of the same software, employing default settings. The significance of the TF activities was assessed, sorted by p‐value, and the top five transcription factors for each organ were again selected for representation on dot plots.

### Nanopore Direct RNA Sequencing (DRS)

The DRS was performed following the protocol previously described.^[^ ^6^ ^]^ Initially, 75 µg of RNA, isolated via the Trizol method, was diluted to 100 µL with nuclease‐free water. The polyadenylated RNA species were then selectively extracted using the Dynabeads mRNA Purification Kit (Invitrogen, Cat. 61 006) as per the manufacturer's guidelines. Once purified, the polyadenylated RNAs were resuspended in nuclease‐free water. The quality and quantity of the RNAs were evaluated using the NanoDrop 2000 spectrophotometer (Thermo Fisher Scientific). For sequencing, 500 ng of the isolated polyadenylated RNAs were prepared for Nanopore direct RNA sequencing following the protocol of the Oxford Nanopore Technologies (ONT) SQK‐RNA002 kit, including an optional reverse transcription step as recommended by ONT. The libraries were loaded onto ONT R9.4 flow cells and sequenced on the GridION platform using the standard MinKNOW/19.12.5 protocol script.

### Isoform‐Identification of DRS

The analysis flow was performed as previously described.^[^ ^21^, ^45^ ^]^ The ONT Guppy software/2.1.0 served as the basecalling platform for the DRS data, converting raw signals into readable sequences. Subsequently, the generated FASTQ files were aligned to the GRCm38 mouse genome using minimap2 /2.17, with specific parameters (“‐ax splice ‐uf ‐k14”) optimized for splice alignment. Further analysis was carried out using the FLAIR pipeline (https://flair.readthedocs.io/en/latest/), with certain adaptations to enhance isoform detection. The process began by excluding reads that exhibited deletion lengths greater than 100 nucleotides. Only reads whose 5′ ends overlapped with chromatin open regions, as identified by ATAC‐Seq data, were considered for further analysis. The accuracy of splice‐site boundaries in DRS reads was improved using the FLAIR “correct” module by aligning them with corresponding short‐read splice junctions. This module applies a default 15 base pair threshold for validating long‐read splice sites, meaning that a splice site from long‐read data is considered valid if a corresponding splice junction from short‐read data is found within ±15 base pairs. A splice junction was only accepted if it was supported by at least three uniquely mapping short reads, ensuring the reliability of the splicing data. The final step in the analysis involved using the default settings of the FLAIR “collapse” function to compile and annotate the DRS isoforms across all organs or targeting a specific organ.

### Isoform Comparison

The reference annotations for the analysis were sourced from the GENCODE database (GENCODE vM24). DRS data and these public reference annotations were compared using GffCompare, available at https://github.com/gpertea/gffcompare, employing default settings as cited in reference.^[^ ^8^ ^]^ The comparison outputs were summarized in a GTF file, where the “class code” attribute was crucial for categorizing the transcripts/isoforms. Transcripts that received a class code of “ = ” were identified as matched transcripts, indicating their intron chains were exactly consistent with those in the reference annotations. Conversely, transcripts assigned class codes of “m, j, o, x, i, y, or u” were considered novel or different, signifying variations in isoform structures compared to the reference. These codes indicate varying degrees of discrepancy from the reference, such as alternative splicing or intron retention. Transcripts classified with codes “c, k, n, e, s, p, or r” were labeled as “others”, characterized by partially matching splicing sites or no direct overlap with the reference annotation, indicating partial similarities or significant discrepancies. Particularly, transcripts with a “u” class code were categorized as representing novel genes, as they showed no overlap with any existing genes in the reference database (GENCODE vM24). Additionally, by comparing annotations across different tissues, tissue‐specific transcripts were defined as those that differ from transcripts in any other tissues.

### Transcript Differential Expression and Alternative Splicing Analyses of DRS

The quantification of Nanopore reads relative to the DRS annotations was conducted using the FLAIR “quantify” function under default parameters. For this process, only alignments with quality scores of 1 or greater were considered for counting. Differential transcript expression analysis was then performed utilizing Fisher's exact test to determine statistically significant changes between conditions. Expression levels were normalized to counts per million (CPM), and the fold change (FC) between fasting and fed conditions was calculated as (CPM _fasting_+0.01)/ (CPM _fed_+0.01). A threshold for significant differential expression was established at a log2(FC) greater than 0.5 and with a p‐value of less than 0.01. In addition to differential expression, the FLAIR “diffSplice” function was used to identify four alternative splicing events—Intron Retention (IR), Alternative 3′ Splicing (ALT3), Alternative 5′ Splicing (ALT5), and Exon Skipping (ES)—using the default settings. The SUPPA2 pipeline (https://github.com/comprna/SUPPA) was also employed to characterize additional splicing types, including Alternative First Exons (AF), Alternative Last Exons (AL), and Mutually Exclusive Exons (MX).^[^ ^46^, ^47^, ^48^, ^49^ ^]^ The analysis of these AS events was further refined using “diffsplice_fishers_exact”, which applies Fisher's exact test to assess significance. AS events with a p‐value less than 0.01 were considered significantly altered.

### Protein Structure Prediction, Visualization and Function Prediction

The putative peptide sequences for the transcripts were initially predicted using CPC2 (http://cpc2.gao‐lab.org/index.php) with default parameters.^[^ ^50^ ^]^ Following this, the predicted sequences were subjected to structural modeling using the AlphaFold/2.1.2 (https://github.com/deepmind/alphafold),^[^ ^51^ ^]^ also utilizing default settings. For functional insights into the modeled proteins, I‐TASSER was employed to predict potential ligand binding sites along with the confidence scores (C‐scores) of these predictions.^[^ ^52^ ^]^ All top‐ranked structural models were visualized and compared using ChimeraX version 1.7.

### Gene Ontology (GO), Gene Set Variation Analysis (GSVA) and Gene Set Enrichment Analysis (GSEA) Pathway Enrichment Analyses

The GO term enrichment analysis for Biological Processes (BP) was conducted using the R package clusterProfiler/3.18.0 (https://github.com/YuLab‐SMU/clusterProfiler), applied with its default settings. Gene expression data from both short‐read RNA‐Seq and Nanopore DRS for the fed samples were processed using the R package GSVA/1.38.0 (https://github.com/rcastelo/GSVA),^[^ ^53^ ^]^ which estimates the enrichment signatures of pathways for each sample, providing a non‐parametric, unsupervised method for assessing pathway activity from gene expression data. The resulting GSVA enrichment scores were normalized using z‐score transformation and visualized using the R package pheatmap/1.0.12. Additionally, GSEA analysis was performed using WebGestalt with default settings, based on the sorted gene lists according to their fold changes.^[^ ^54^ ^]^

### Poly(A) Tail Length Analysis of DRS

The estimation of poly(A) tail lengths from DRS data was conducted using the pipeline‐poly(A)‐ng,^[^ ^21^ ^]^ available at https://github.com/nanoporetech/pipeline‐polya‐ng. This tool utilized the transcript sequence data derived from the DRS isoform identification phase as the reference transcriptome. To ensure the reliability of the analysis, reads with a mapping quality below 5 were excluded, while other settings were retained at default values. Further analysis to assess changes in poly(A) tail lengths between different treatment conditions was performed using pipeline‐poly(A)‐diff, accessible at https://github.com/nanoporetech/pipeline‐polya‐diff. This tool compared poly(A) tail lengths across treatments using default parameter settings. Significant shifts in poly(A) tail lengths were determined based on a cutoff of length changes greater than 10 nucleotides and a p‐value less than 0.05.

### m6A Modification Identification and Analysis

The analysis of RNA m6A modifications in Direct RNA Sequencing (DRS) data was conducted using the MINES tool (https://github.com/YeoLab/MINES).^[^ ^55^ ^]^ Initially, DRS reads and their associated modification values were processed using Tombo (https://github.com/nanoporetech/tombo), aligning to a genomic reference (GRCm38/mm10 from GENCODE) under default settings for re‐squiggling and de novo modification detection. To focus on potential m6A sites, a new set of genomic regions was created by extending 10 base pairs on either side of the “A” in the DRACH motifs, which are typically associated with m6A modifications. This extension ensured comprehensive coverage of all relevant areas in the genome containing DRACH motifs. These areas were further filtered to require a minimum coverage of five reads to ensure data reliability. Modification events occurring in fewer than two of the total samples were excluded to minimize false positives. The spatial distribution and aggregation of the modification sites across the genome were analyzed using MetaPlotR (https://github.com/olarerin/metaPlotR) with default settings.^[^ ^56^ ^]^ Briefly, a region annotation file was created to catalog transcript region coordinates (i.e., 5'UTR, CDS, and 3'UTR) using the “size_of_cds_utrs” module with default settings on protein coding genes, as specified by GENCODE vM24 reference annotation obtained from the UCSC Table Browser (http://genome.ucsc.edu/cgi‐bin/hgTables). The “rel_and_abs_dist_calc” module was then applied to characterize m6A modification loci within gene bodies according to this region annotation. The distribution of the modifications in the genome was visualized using the R package karyoploteR/3.19. Differential analysis of m6A modification events was conducted using Fisher's exact test. Significant changes in modifications were identified using a log2 (fold change) cutoff of greater than 0.5 and a p‐value of less than 0.01, pinpointing regions with significant alterations in m6A modification between conditions.

### Principal Component Analysis (PCA)

The DESeq2/3.1.0 package in R was employed for the principal component analysis (PCA) of alternative splicing events and m6A modification coverages. To prepare the data for PCA, the “rlog” function within DESeq2 was used to regularize the log transformation of the data. This regularization step helps to stabilize the variance across the range of values, making the data more amenable for PCA by reducing the influence of extreme values. Following data regularization, the “plotPCA” function from DESeq2 was utilized to perform the PCA. This analysis primarily focuses on reducing the dimensionality of the data, allowing for the exploration of the major patterns of variation and highlighting the most significant components. The results were visualized by plotting the first two principal components, which often capture the largest variation in the dataset. Samples in the PCA plot were color‐coded according to a specified grouping variable, facilitating easy visualization and interpretation of the data clustering and differences based on predefined groups or conditions.

### Statistics

Comprehensive statistical details of the experiments, data analysis, and presentation are provided in the figure legends and the manuscript above. Differential transcript expression analysis was conducted using Fisher’s exact test to identify statistically significant changes between conditions. Analysis of alternative splicing events was performed with the FLAIR “diffsplice_fishers_exact” module, while changes in poly(A) tail lengths between treatment conditions were assessed using the pipeline‐poly(A)‐diff tool (https://github.com/nanoporetech/pipeline‐polya‐diff). Differential analysis of m6A modification events was carried out using Fisher’s exact test. For comparisons between two groups, a two‐tailed unpaired Student's t‐test was applied. All statistical analyses were executed using R (v4.4.1) or Python (v3.12.4). Unless otherwise specified, a p‐value of less than 0.05 was considered statistically significant.

### Study Approval

All animal experiments were conducted following the guidelines approved by the National Heart, Lung, and Blood Institute (NHLBI) Animal Care Committee.

## Conflict of Interest

The authors declare no conflict of interest.

## Supporting information



Supporting Information

Supplemental Tables

## Data Availability

The source data underlying the figures, supplementary tables, and supplementary figures are provided as a Source Data file. The raw sequencing data can be accessed at GEO through the accession numbers: GSE267280 for RNA‐Seq data (reviewer private token: cjmpamukpbmtdib), GSE267279 for ATAC‐Seq data (reviewer private token: mbwfcosqrbsphix), and GSE267281 for nanopore direct RNA sequencing data (reviewer private token: ylezosuchxqvnwv). All data is available from the corresponding author upon reasonable request.
