# Supplementary material for: Unveiling Tissue‐Specific RNA Landscapes in Mouse Organs During Fasting and Feeding Using Nanopore Direct RNA Sequencing
Source: Adv Sci (Weinh). 2024 Dec 16;12(5):2408054. doi: 10.1002/advs.202408054 (PMC11792027; doi:10.1002/advs.202408054)
Supplement: Supplementary file 1 — Supporting Information [file ADVS-12-2408054-s002.pdf]

ADVANCED SCIENCE
Open Access

Supporting Information

for *Adv. Sci.*, DOI 10.1002/advs.202408054

Unveiling Tissue-Specific RNA Landscapes in Mouse Organs During Fasting and Feeding Using Nanopore Direct RNA Sequencing

*Chengfei Jiang, Ping Li and Haiming Cao**

Supporting Information

Unveiling Tissue-Specific RNA Landscapes in Mouse Organs During Fasting and Feeding Using Nanopore Direct RNA Sequencing

*Chengfei Jiang, Ping Li and Haiming Cao**

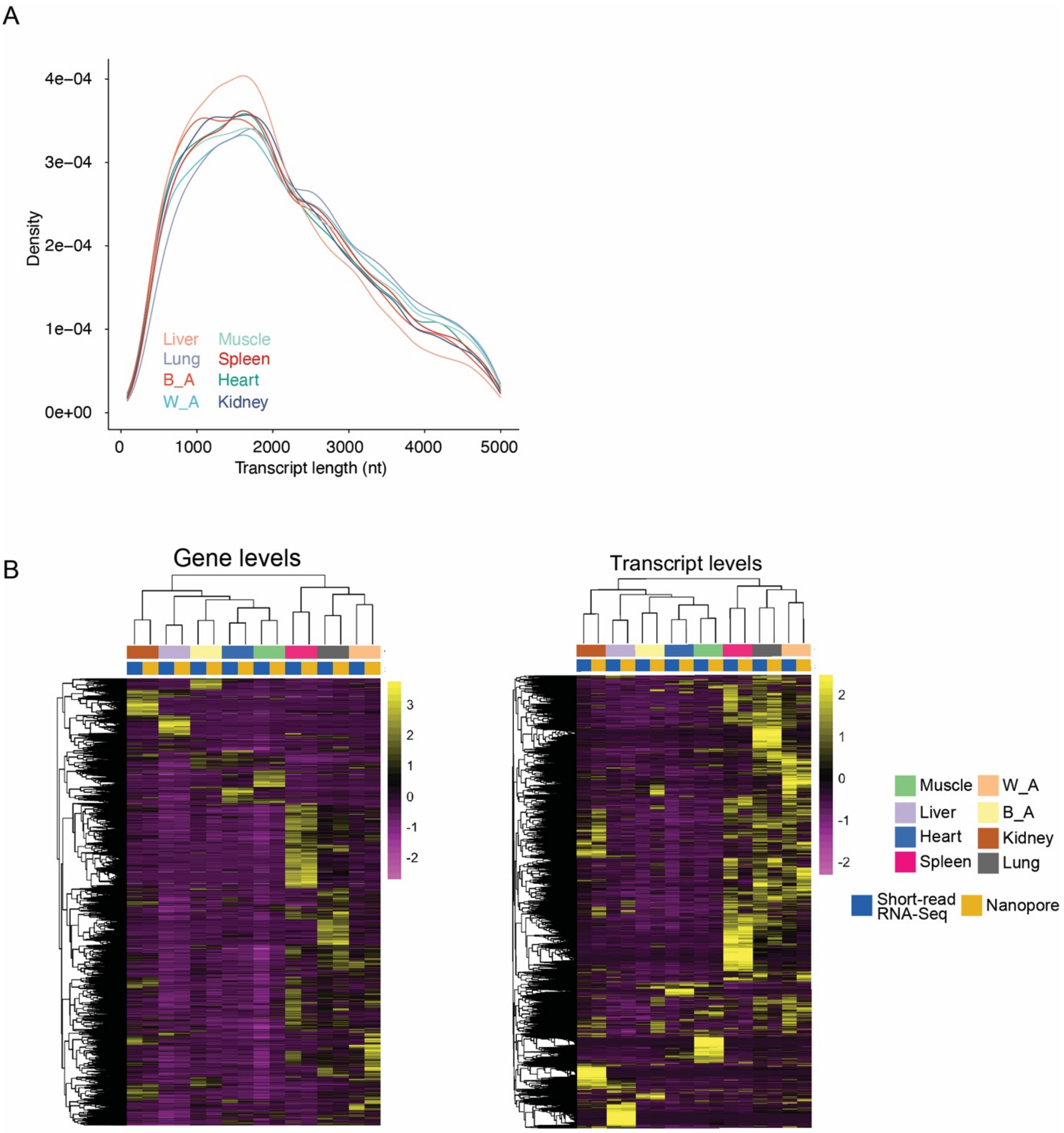

Figure S1 Tissue-specific RNA landscapes revealed by Nanopore direct RNA sequencing (DRS)

(A) Length distribution of DRS transcripts ranging from 0 to 5000 nucleotides in different tissues. (B) Heatmap displaying gene-level (left) and transcript-level (right) expression data from different fed tissue samples, derived from both short-read RNA-Seq and DRS.

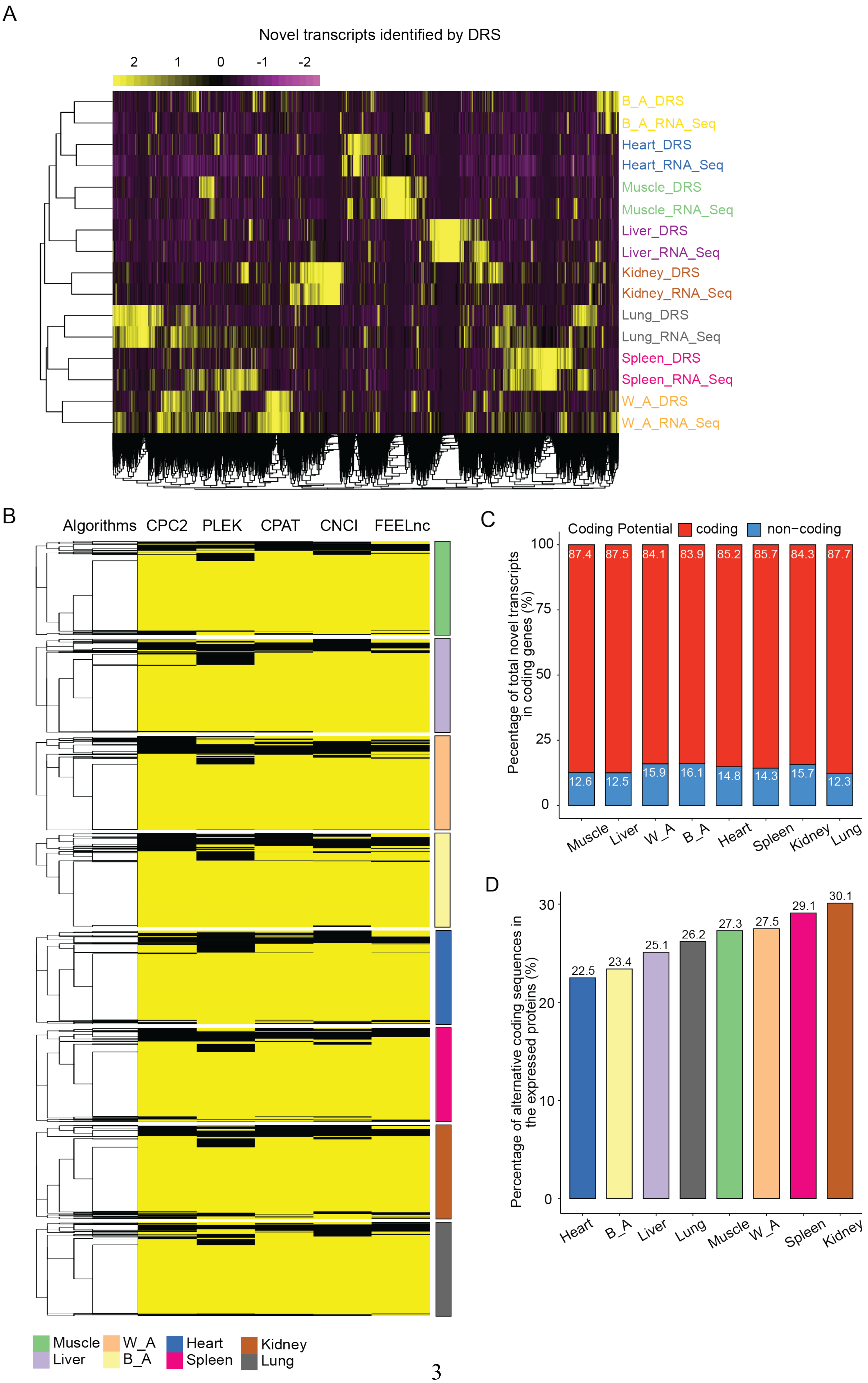
A
Novel transcripts identified by DRS
2 1 0 -1 -2
B_A_DRS
B_A_RNA_Seq
Heart_DRS
Heart_RNA_Seq
Muscle_DRS
Muscle_RNA_Seq
Liver_DRS
Liver_RNA_Seq
Kidney_DRS
Kidney_RNA_Seq
Lung_DRS
Lung_RNA_Seq
Spleen_DRS
Spleen_RNA_Seq
W_A_DRS
W_A_RNA_Seq
B
Algorithms CPC2 PLEK CPAT CNCI FEELnc
Muscle W_A Heart Kidney
Liver B_A Spleen Lung
C
Coding Potential coding non−coding
Pecentage of total novel transcripts in coding genes (%)
100 75 50 25 0
87.4 87.5 84.1 83.9 85.2 85.7 84.3 87.7
12.6 12.5 15.9 16.1 14.8 14.3 15.7 12.3
Muscle Liver W_A B_A Heart Spleen Kidney Lung
D
Percentage of alternative coding sequences in the expressed proteins (%)
30 20 10 0
22.5 23.4 25.1 26.2 27.3 27.5 29.1 30.1
Heart B_A Liver Lung Muscle W_A Spleen Kidney

Figure S2 DRS identifies novel transcripts in mouse tissues

(A) Heatmap displaying the expression levels of novel transcripts identified by DRS and short-read RNA-Seq across different tissues in the fed groups. Short-read RNA-Seq reads were quantified using the Salmon pipeline (https://github.com/COMBINE-lab/salmon). Transcript per million (TPM) values from both DRS and short-read RNA-Seq were normalized using Z-score normalization across all transcripts. Each row corresponds to a novel transcript identified by DRS, and each column represents samples from DRS and short-read RNA-Seq. Samples from the same tissue are color-coded consistently to facilitate comparison across platforms. (B) Heatmap of the coding potential analysis results for novel or differing transcripts from protein-coding regions across various tissues. Five coding potential algorithms (CPC2, PLEK, CPAT, CNCI, FEELnc) were employed to evaluate these transcripts. Positive coding hints are marked in yellow. Transcripts were defined as coding if they met the following criteria: CPC2 (score > 0.5, http://cpc2.gao-lab.org/index.php), CPAT (score > 0.36, http://lilab.research.bcm.edu/cpat/index.php), CNCI (score > 0, https://github.com/www-bioinfo-org/CNCI), FEELnc (default parameters, https://github.com/tderrien/FEELnc), and PLEK (score > 0, https://sourceforge.net/projects/plek/files/). (C) Bar plot representing the percentage of predicted coding versus non-coding transcripts among all novel transcripts derived from coding genes across different tissues. Only novel transcripts identified as coding by at least three of the algorithms were considered coding. (D) Bar plot illustrating the percentage of expressed coding genes exhibiting alternative coding sequences. Only genes with alternative coding sequences different from the reference annotation (GENCODE vM24, https://ftp.ebi.ac.uk/pub/databases/gencode/Gencode_mouse/release_M24/gencode.vM24.pc_translations.fa.gz) were included.

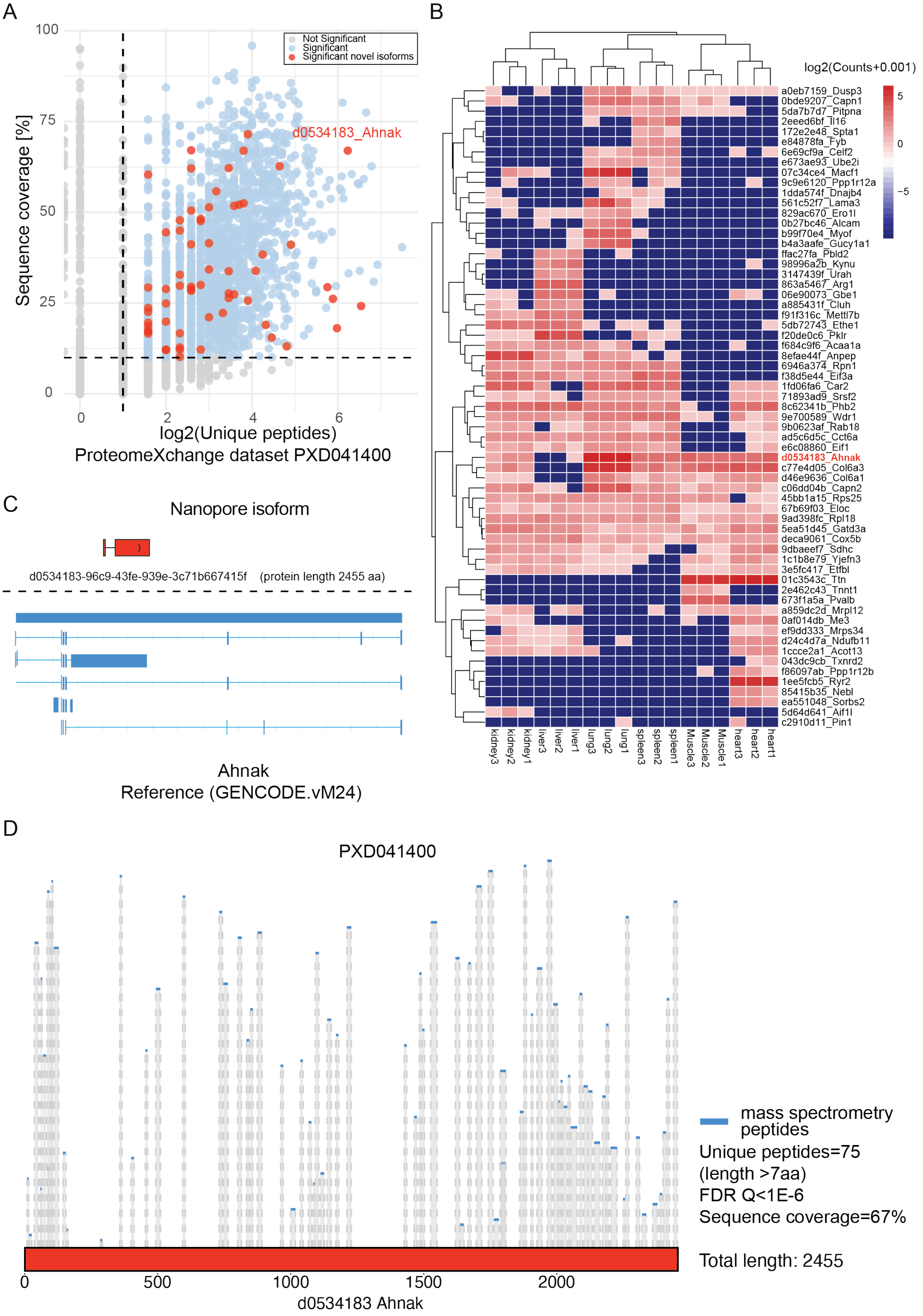
A
100
75
50
25
0
Sequence coverage [%]
0
2
4
6
log2(Unique peptides)
ProteomeXchange dataset PXD041400
Not Significant
Significant
Significant novel isoforms
d0534183_Ahnak
B
log2(Counts+0.001)
5
0
−5
a0eb7159_Dusp3
0bde9207_Capn1
5da7b7d7_Pitpna
2eeed6bf_Il16
172e2e48_Spta1
e84878fa_Fyb
6e69cf9a_Celf2
e673ae93_Ube2i
07c34ce4_Macf1
9c9e6120_Ppp1r12a
1dda574f_Dnajb4
561c52f7_Lama3
829ac670_Ero1l
0b27bc46_Alcam
b99f70e4_Myof
b4a3aafe_Gucy1a1
ffac27fa_Pbld2
98996a2b_Kynu
3147439f_Urah
863a5467_Arg1
06e90073_Gbe1
a885431f_Cluh
f91f316c_Mettl7b
5db72743_Ethe1
f20de0c6_Pklr
f684c9f6_Acaa1a
8efae44f_Anpep
6946a374_Rpn1
f38d5e44_Eif3a
1fd06fa6_Car2
71893ad9_Srsf2
8c62341b_Phb2
9e700589_Wdr1
9b0623af_Rab18
ad5c6d5c_Cct6a
e6c08860_Eif1
d0534183_Ahnak
c77e4d05_Col6a3
d46e9636_Col6a1
c06dd04b_Capn2
45bb1a15_Rps25
67b69f03_Eloc
9ad398fc_Rpl18
5ea51d45_Gatd3a
deca9061_Cox5b
9dbaeef7_Sdhc
1c1b8e79_Yjefn3
3e5fc417_Etfbl
01c3543c_Ttn
2e462c43_Tnnt1
673f1a5a_Pvalb
a859dc2d_Mrpl12
0af014db_Me3
ef9dd333_Mrps34
d24c4d7a_Ndufb11
1ccce2a1_Acot13
043dc9cb_Txnrd2
f86097ab_Ppp1r12b
1ee5fcb5_Ryr2
85415b35_Nebl
ea551048_Sorbs2
5d64d641_Aif1l
c2910d11_Pin1
kidney3
kidney2
kidney1
liver3
liver2
liver1
lung3
lung2
lung1
spleen3
spleen2
spleen1
Muscle3
Muscle2
Muscle1
heart3
heart2
heart1
C
Nanopore isoform
d0534183-96c9-43fe-939e-3c71b667415f (protein length 2455 aa)
Ahnak
Reference (GENCODE.vM24)
D
PXD041400
mass spectrometry peptides
Unique peptides=75
(length >7aa)
FDR Q<1E-6
Sequence coverage=67%
Total length: 2455
0
500
1000
1500
2000
d0534183 Ahnak

Figure S3 Validation of novel transcripts in the proteome dataset

(A) Scatter plot illustrating the relationship between log2(Unique peptides) and sequence coverage [%] across the proteome dataset (PXD041400). The data was obtained from ProteomeXchange (PXD041400, https://proteomecentral.proteomexchange.org/cgi/GetDataset?ID=PXD041400), consisting of three samples for each organ (heart, lung, liver, kidney, spleen, and muscle) from 8-week-old male mice. Each point represents a protein, color-coded according to filter categories: "Significant & Yes" (red), "Significant" (light blue), and "Not Significant" (gray). A horizontal dashed line marks a sequence coverage of 10%, while a vertical dashed line marks log2(2) unique peptides (corresponding to Unique peptides > 2). The protein "d0534183_Ahnak" (d0534183-96c9-43fe-939e-3c71b667415f) is highlighted in red, with the label positioned above the point. (B) Heatmap showing the log2-transformed unique peptide counts across various conditions for distinct proteins. This provides a visual comparison of protein abundance across the different samples. (C) Schematic representation of the novel transcript identified by Direct RNA Sequencing (DRS) for the Ahnak gene (d0534183-96c9-43fe-939e-3c71b667415f, d0534183_Ahnak, in red) compared to the reference annotation (in blue), highlighting differences between the novel and annotated transcripts in the gene body. (D) Peptide alignment visualization for each peptide identified in the d0534183_Ahnak protein in the proteome dataset PXD041400. The reference protein sequence is represented by a red horizontal bar, while the aligned peptides are shown as blue rectangles above the reference. The peptides are staggered along the y-axis to avoid overlap, and shaded gray areas with dashed lines indicate the coverage range of each peptide.

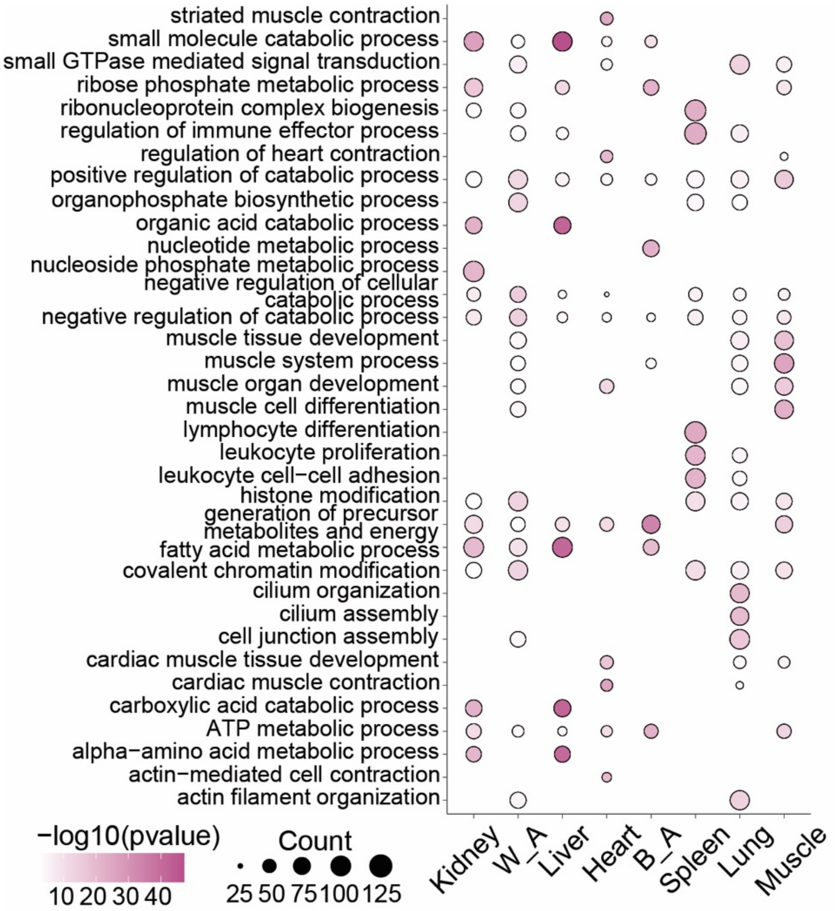

Figure S4 Pathway analysis of tissue-specific transcripts

Pathway enrichment analysis (GO term, Biological Process) for tissue-specific transcripts. The top five enriched pathways, based on the p-value, were selected for each tissue.

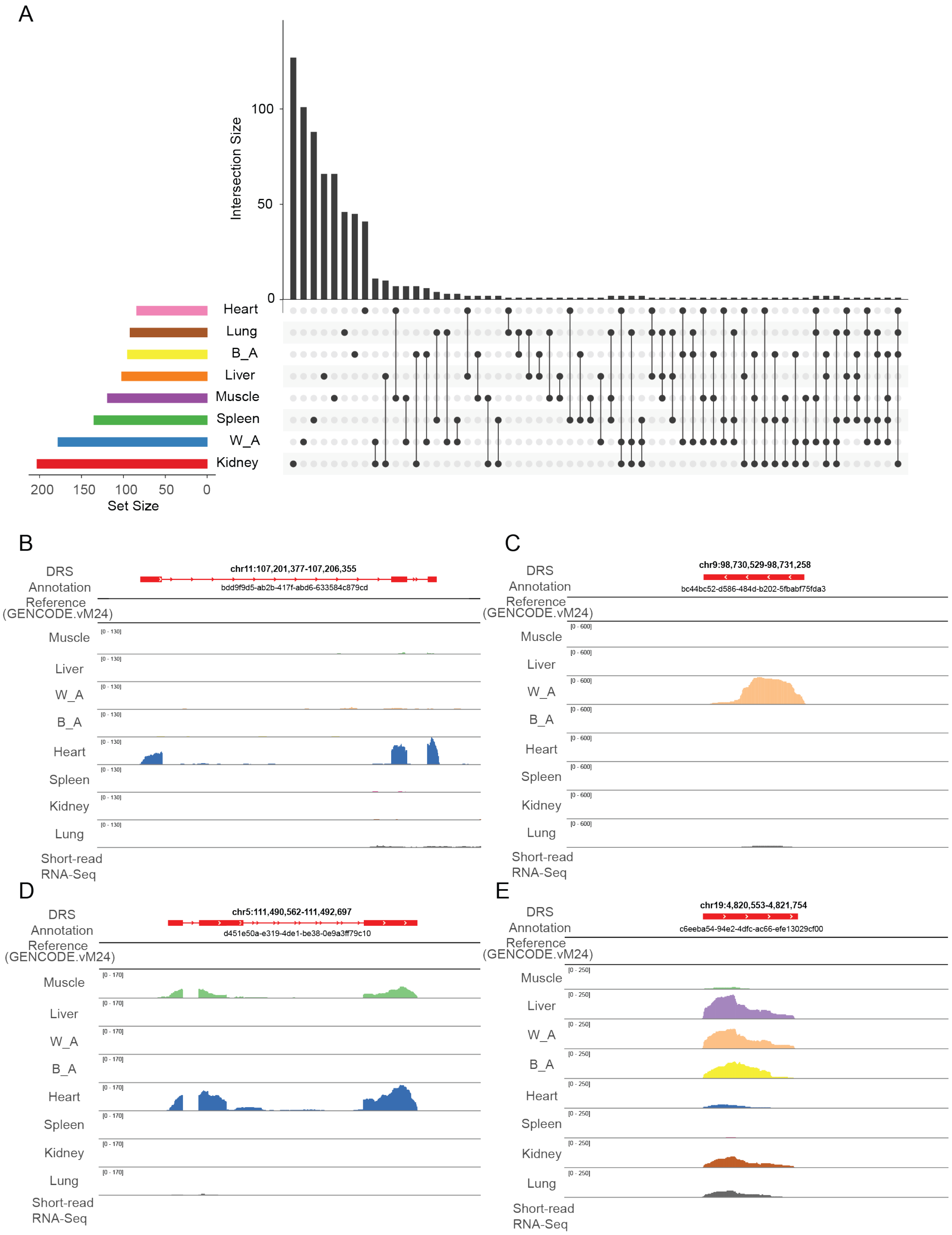

Figure S5 DRS discovers novel genes in mouse tissues

(A) UpSet plot of the intersections of novel genes identified in different tissues. (B-E) Top: Isoform schematics of novel gene transcripts for the following identifiers: bdd9f9d5-ab2b-417f-abd6-633584c879cd (B), bc44bc52-d586-484d-b202-5fbabf75fda3 (C), d451e50a-e319-4de1-be38-0e9a3ff79c10 (D), and c6eeba54-94e2-4dfc-ac66-efe13029cf00 (E).Bottom: The short-

read RNA-Seq read coverage from various tissues is visualized using IGV (Integrative Genomics Viewer) and displayed in different colors to indicate tissue-specific coverage.

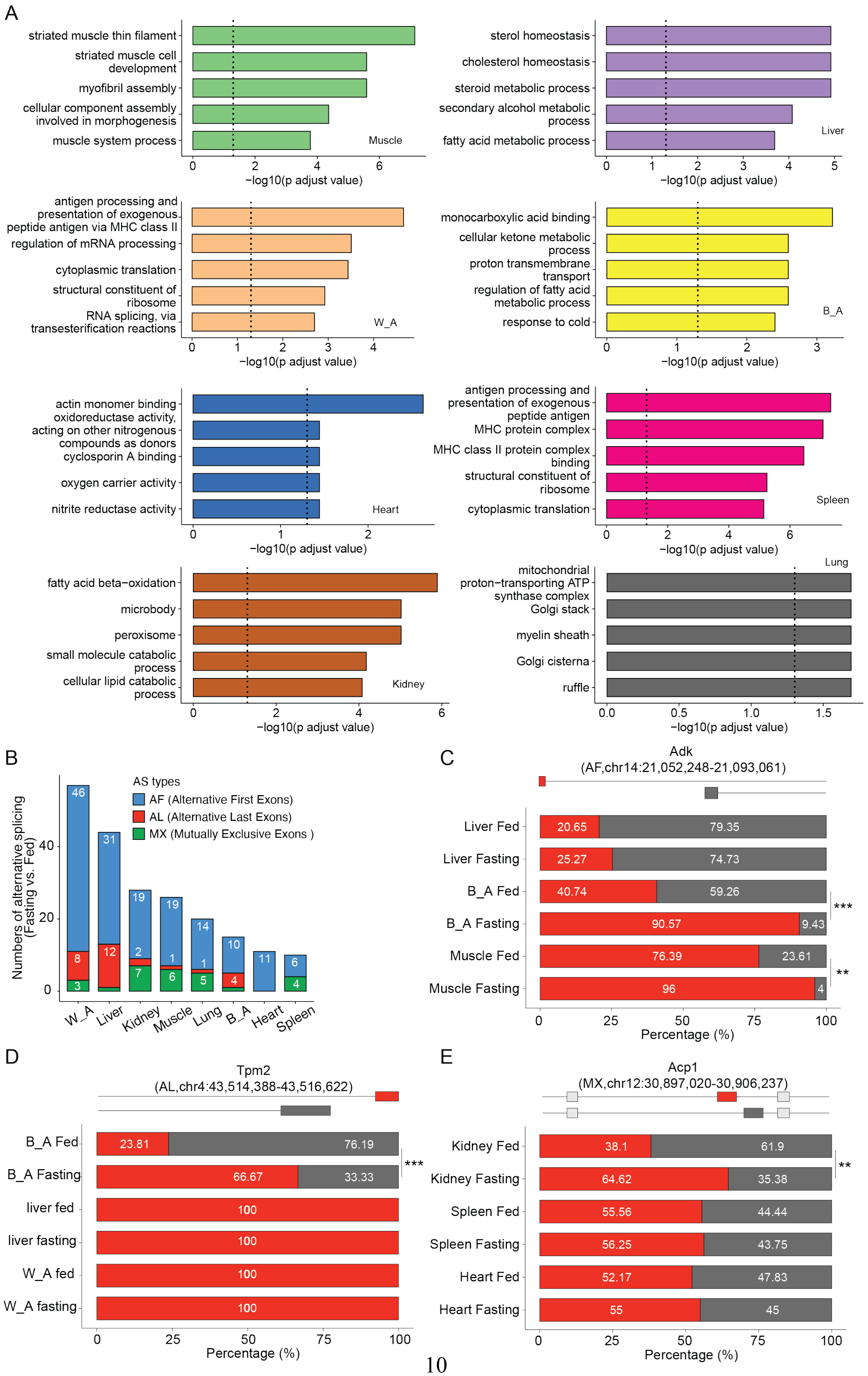
A
striated muscle thin filament
striated muscle cell development
myofibril assembly
cellular component assembly involved in morphogenesis
muscle system process
Muscle
0 2 4 6
−log10(p adjust value)
sterol homeostasis
cholesterol homeostasis
steroid metabolic process
secondary alcohol metabolic process
fatty acid metabolic process
Liver
0 1 2 3 4 5
−log10(p adjust value)
antigen processing and presentation of exogenous peptide antigen via MHC class II
regulation of mRNA processing
cytoplasmic translation
structural constituent of ribosome
RNA splicing, via transesterification reactions
W_A
0 1 2 3 4
−log10(p adjust value)
monocarboxylic acid binding
cellular ketone metabolic process
proton transmembrane transport
regulation of fatty acid metabolic process
response to cold
B_A
0 1 2 3
−log10(p adjust value)
actin monomer binding
oxidoreductase activity, acting on other nitrogenous compounds as donors
cyclosporin A binding
oxygen carrier activity
nitrite reductase activity
Heart
0 1 2
−log10(p adjust value)
antigen processing and presentation of exogenous peptide antigen
MHC protein complex
MHC class II protein complex binding
structural constituent of ribosome
cytoplasmic translation
Spleen
0 2 4 6
−log10(p adjust value)
fatty acid beta−oxidation
microbody
peroxisome
small molecule catabolic process
cellular lipid catabolic process
Kidney
0 2 4 6
−log10(p adjust value)
Lung
mitochondrial proton−transporting ATP synthase complex
Golgi stack
myelin sheath
Golgi cisterna
ruffle
0.0 0.5 1.0 1.5
−log10(p adjust value)
B
Numbers of alternative splicing (Fasting vs. Fed)
AS types
AF (Alternative First Exons)
AL (Alternative Last Exons)
MX (Mutually Exclusive Exons)
46 8 3
31 12
19 2 7
19 1 6
14 1 5
10 4
11
6 4
0 20 40
W_A Liver Kidney Muscle Lung B_A Heart Spleen
C
Adk
(AF,chr14:21,052,248-21,093,061)
Liver Fed 20.65 79.35
Liver Fasting 25.27 74.73
B_A Fed 40.74 59.26
B_A Fasting 90.57 9.43
Muscle Fed 76.39 23.61
Muscle Fasting 96 4
***
**
0 25 50 75 100
Percentage (%)
D
Tpm2
(AL,chr4:43,514,388-43,516,622)
B_A Fed 23.81 76.19
B_A Fasting 66.67 33.33
liver fed 100
liver fasting 100
W_A fed 100
W_A fasting 100
***
0 25 50 75 100
Percentage (%)
E
Acp1
(MX,chr12:30,897,020-30,906,237)
Kidney Fed 38.1 61.9
Kidney Fasting 64.62 35.38
Spleen Fed 55.56 44.44
Spleen Fasting 56.25 43.75
Heart Fed 52.17 47.83
Heart Fasting 55 45
**
0 25 50 75 100
Percentage (%)

Figure S6 DRS reveals alternative splicing alterations during fasting treatments

(A) Bar plot showing the -log10 (adjusted p-value) of the top 5 significantly enriched pathways across different tissues. Each bar represents a pathway, with its height indicating statistical significance (-log10 of the adjusted p-value). Genes with significantly altered splicing events during fasting treatments in each tissue were analyzed through Gene Ontology (GO) term pathway analysis and ranked based on adjusted p-values. (B) Bar plot displaying the numbers of significantly changed alternative splicing (AS) events between fasting and fed conditions in different tissues. AS events with a p-value less than 0.01 were considered to be significantly altered, and different AS types are distinguished by different colors. (C-E) Top: Schematic diagrams illustrating alternative splicing events for the following genes: Adk alternative first exons (C), Tpm2 alternative last exons (D), and Acp1 mutually exclusive exons (E). The gray isoform represents exon exclusion, while the red isoform represents exon inclusion. Bottom: Bar plots showing the proportions of exon exclusion (gray) and exon inclusion (red) events in the specified regions across different tissues for both fed and fasting treatment groups.

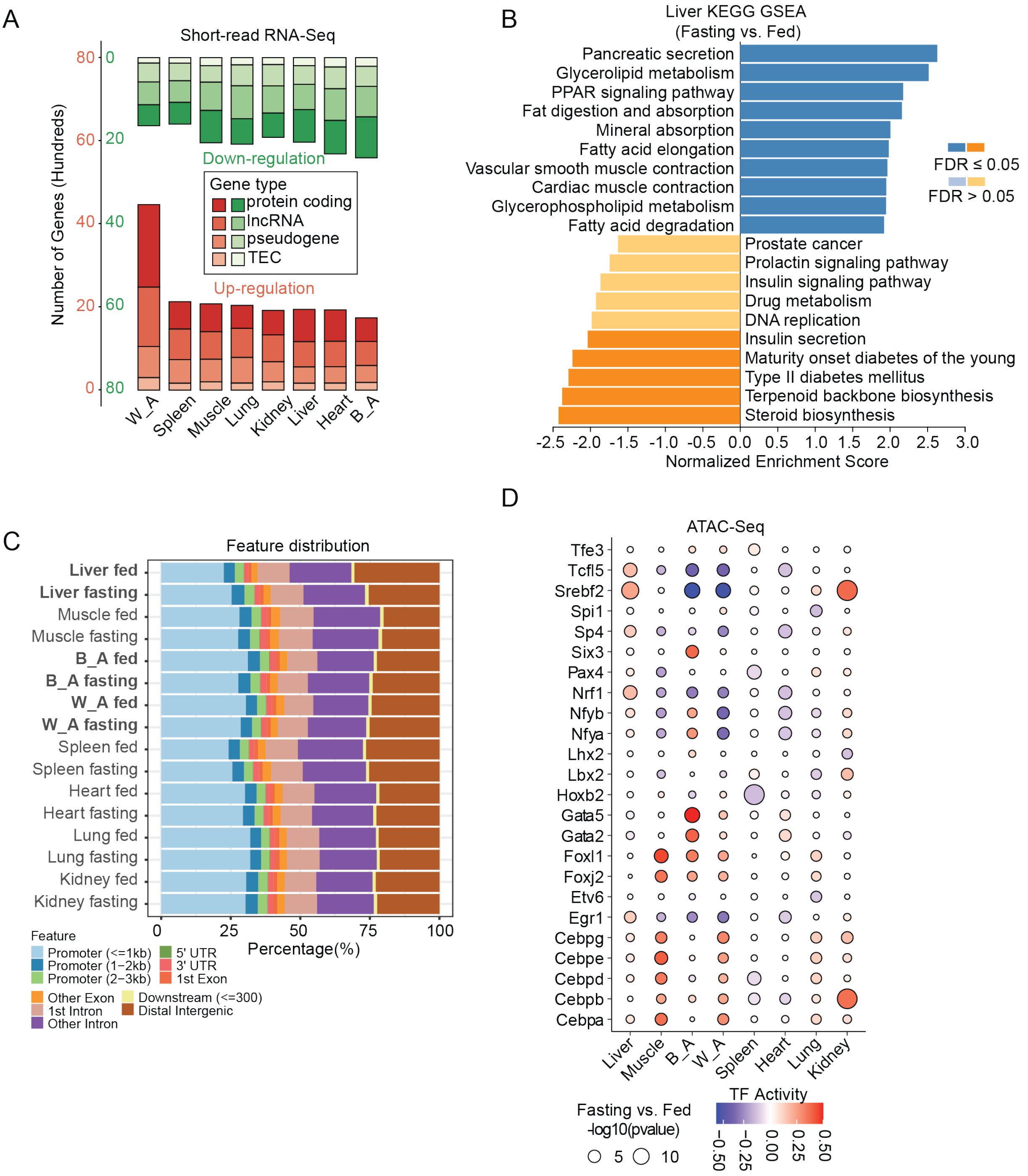
A
Short-read RNA-Seq
Number of Genes (Hundreds)
80
60
40
20
0
0
20
40
60
80
Down-regulation
Up-regulation
Gene type
protein coding
lncRNA
pseudogene
TEC
W_A
Spleen
Muscle
Lung
Kidney
Liver
Heart
B_A
B
Liver KEGG GSEA
(Fasting vs. Fed)
Pancreatic secretion
Glycerolipid metabolism
PPAR signaling pathway
Fat digestion and absorption
Mineral absorption
Fatty acid elongation
Vascular smooth muscle contraction
Cardiac muscle contraction
Glycerophospholipid metabolism
Fatty acid degradation
Prostate cancer
Prolactin signaling pathway
Insulin signaling pathway
Drug metabolism
DNA replication
Insulin secretion
Maturity onset diabetes of the young
Type II diabetes mellitus
Terpenoid backbone biosynthesis
Steroid biosynthesis
FDR ≤ 0.05
FDR > 0.05
-2.5 -2.0 -1.5 -1.0 -0.5 0.0 0.5 1.0 1.5 2.0 2.5 3.0
Normalized Enrichment Score
C
Feature distribution
Liver fed
Liver fasting
Muscle fed
Muscle fasting
B_A fed
B_A fasting
W_A fed
W_A fasting
Spleen fed
Spleen fasting
Heart fed
Heart fasting
Lung fed
Lung fasting
Kidney fed
Kidney fasting
0 25 50 75 100
Percentage(%)
Feature
Promoter (<=1kb)
Promoter (1−2kb)
Promoter (2−3kb)
5' UTR
3' UTR
1st Exon
Other Exon
1st Intron
Other Intron
Downstream (<=300)
Distal Intergenic
D
ATAC-Seq
Tfe3
Tcfl5
Srebf2
Spi1
Sp4
Six3
Pax4
Nrf1
Nfyb
Nfya
Lhx2
Lbx2
Hoxb2
Gata5
Gata2
Foxl1
Foxj2
Etv6
Egr1
Cebpg
Cebpe
Cebpd
Cebpb
Cebpa
Liver
Muscle
B_A
W_A
Spleen
Heart
Lung
Kidney
Fasting vs. Fed
-log10(pvalue)
5
10
TF Activity
−0.50
−0.25
0.00
0.25
0.50

Figure S7 Short-read RNA-Seq and ATAC-Seq reveal the gene-level regulation in response to metabolic treatment

(A) Bar plot depicting the number and composition of genes upregulated (red) and downregulated (green) in various tissues, analyzed through short-read RNA-seq under fasting compared to fed conditions. Significant differential genes were determined using Fisher's exact test, with those showing a log2(fold change) greater than 0.5 and a p-value less than 0.01 defined as differentially expressed. Gradient colors represent different gene type compositions. (B) Bar plot of liver Kyoto Encyclopedia of Genes and Genomes (KEGG) pathway Gene Set Enrichment Analysis (GSEA) results using the significantly changed genes during fasting treatment. This plot illustrates the enriched pathways based on their normalized enrichment scores. (C) Distribution of open chromatin loci relative to the transcription start site (TSS) in different tissues and treatments. (D) Transcription factor binding activities at open chromatin loci in each tissue and treatment.

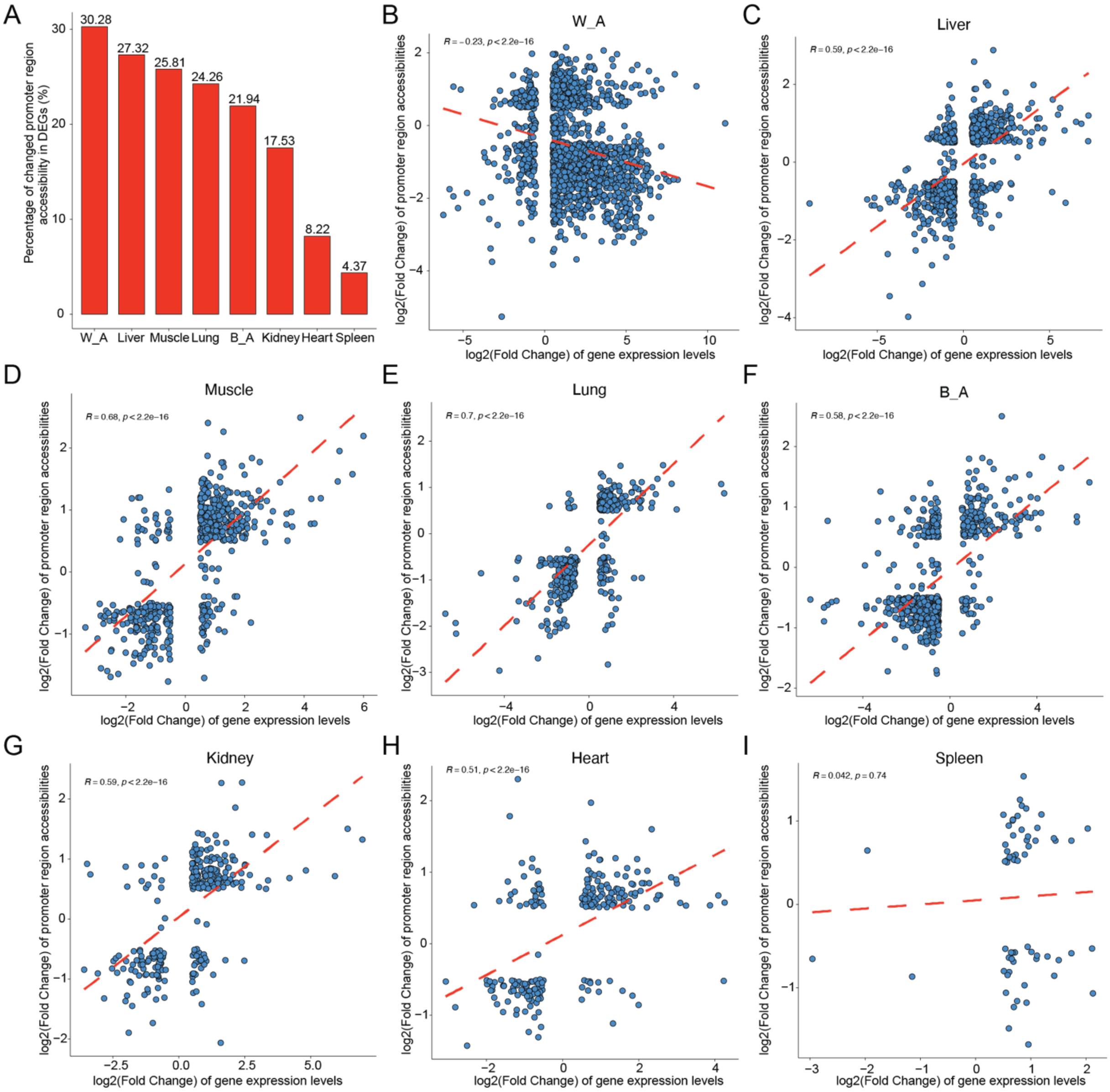
A
Percentage of changed promoter region accessibility in DEGs (%)
30.28
27.32
25.81
24.26
21.94
17.53
8.22
4.37
W_A
Liver
Muscle
Lung
B_A
Kidney
Heart
Spleen
B
W_A
C
Liver
D
Muscle
E
Lung
F
B_A
G
Kidney
H
Heart
I
Spleen
log2(Fold Change) of promoter region accessibilities
log2(Fold Change) of gene expression levels

Figure S8 Association between changes in promoter region accessibility and gene expression levels during fasting treatment

(A) Bar plot showing the percentage of differentially expressed genes (DEGs) during the fasting treatment that also exhibit significantly altered chromatin accessibility in their promoter regions. The promoter regions were defined as the 3000 bp upstream and downstream of the transcription start site (TSS). Only genes and chromatin accessibilities with significant changes ($|\log2(\text{fold change})| > 0.5$ and p-value < 0.01) were included. (B-I) Pearson's correlation analysis comparing the log2(fold change) of DEGs and chromatin accessibilities from different tissues during the fasting treatment.

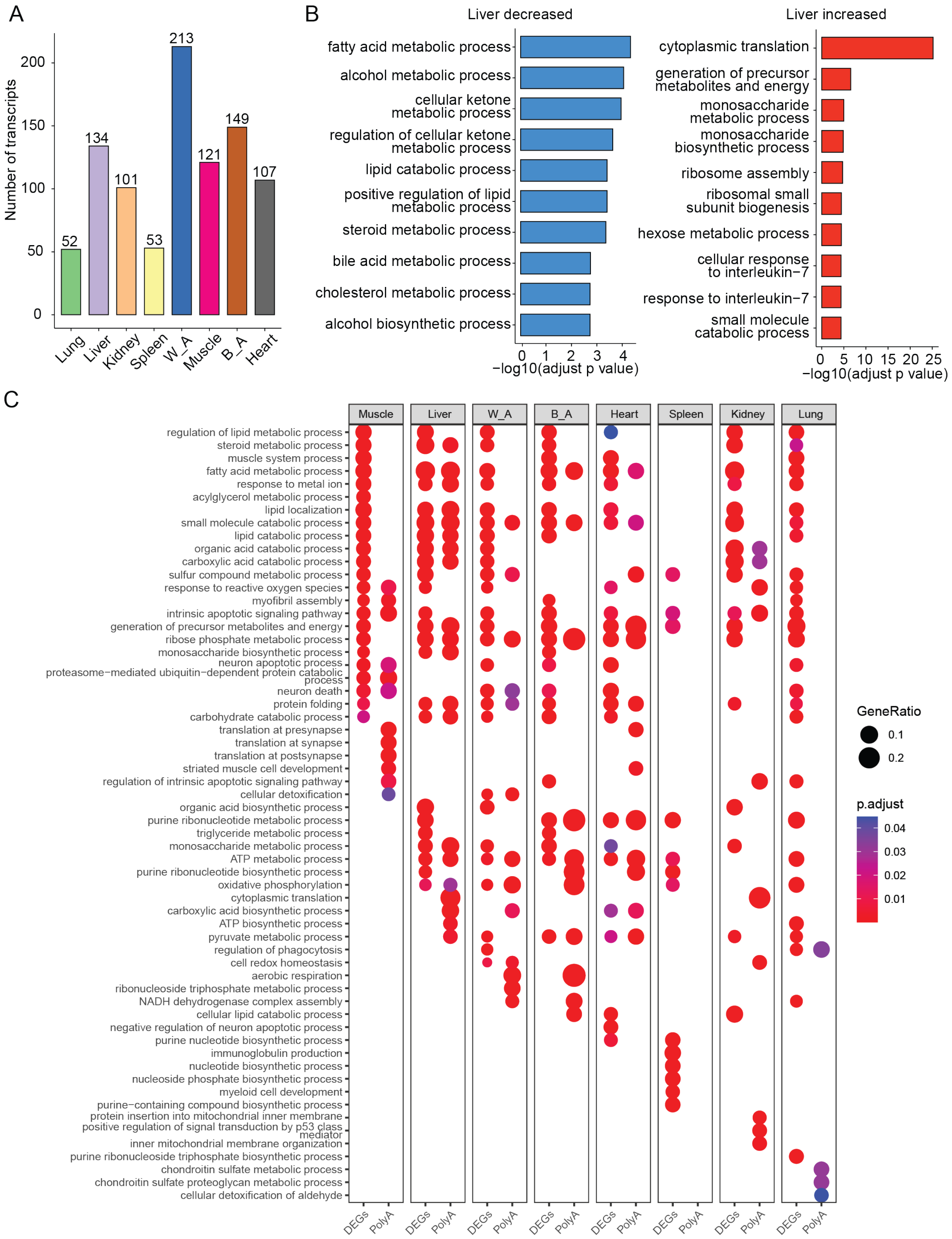

A
Number of transcripts
0
50
100
150
200
52
134
101
53
213
121
149
107
Lung
Liver
Kidney
Spleen
W_A
Muscle
B_A
Heart
B
Liver decreased
fatty acid metabolic process
alcohol metabolic process
cellular ketone metabolic process
regulation of cellular ketone metabolic process
lipid catabolic process
positive regulation of lipid metabolic process
steroid metabolic process
bile acid metabolic process
cholesterol metabolic process
alcohol biosynthetic process
0 1 2 3 4
−log10(adjust p value)
Liver increased
cytoplasmic translation
generation of precursor metabolites and energy
monosaccharide metabolic process
monosaccharide biosynthetic process
ribosome assembly
ribosomal small subunit biogenesis
hexose metabolic process
cellular response to interleukin−7
response to interleukin−7
small molecule catabolic process
0 5 10 15 20 25
−log10(adjust p value)
C
Muscle
Liver
W_A
B_A
Heart
Spleen
Kidney
Lung
regulation of lipid metabolic process
steroid metabolic process
muscle system process
fatty acid metabolic process
response to metal ion
acylglycerol metabolic process
lipid localization
small molecule catabolic process
lipid catabolic process
organic acid catabolic process
carboxylic acid catabolic process
sulfur compound metabolic process
response to reactive oxygen species
myofibril assembly
intrinsic apoptotic signaling pathway
generation of precursor metabolites and energy
ribose phosphate metabolic process
monosaccharide biosynthetic process
neuron apoptotic process
proteasome−mediated ubiquitin−dependent protein catabolic process
neuron death
protein folding
carbohydrate catabolic process
translation at presynapse
translation at synapse
translation at postsynapse
striated muscle cell development
regulation of intrinsic apoptotic signaling pathway
cellular detoxification
organic acid biosynthetic process
purine ribonucleotide metabolic process
triglyceride metabolic process
monosaccharide metabolic process
ATP metabolic process
purine ribonucleotide biosynthetic process
oxidative phosphorylation
cytoplasmic translation
carboxylic acid biosynthetic process
ATP biosynthetic process
pyruvate metabolic process
regulation of phagocytosis
cell redox homeostasis
aerobic respiration
ribonucleoside triphosphate metabolic process
NADH dehydrogenase complex assembly
cellular lipid catabolic process
negative regulation of neuron apoptotic process
purine nucleotide biosynthetic process
immunoglobulin production
nucleotide biosynthetic process
nucleoside phosphate biosynthetic process
myeloid cell development
purine−containing compound biosynthetic process
protein insertion into mitochondrial inner membrane
positive regulation of signal transduction by p53 class mediator
inner mitochondrial membrane organization
purine ribonucleoside triphosphate biosynthetic process
chondroitin sulfate metabolic process
chondroitin sulfate proteoglycan metabolic process
cellular detoxification of aldehyde
DEGs
PolyA
GeneRatio
0.1
0.2
p.adjust
0.04
0.03
0.02
0.01

Figure S9 Polyadenylation variabilities across different tissues

(A) Numbers of transcripts with significantly changed Poly(A) tail lengths. (B) Bar plot showing the top enriched pathways (GO term, Biological Process) for transcripts with significantly decreased or increased Poly(A) tail lengths during fasting treatment in liver tissue. (C) Comparisons of the top enriched pathways (GO term, Biological Process) for DEGs and transcripts with significantly changed Poly(A) tail lengths in different tissues during fasting treatments in DRS.

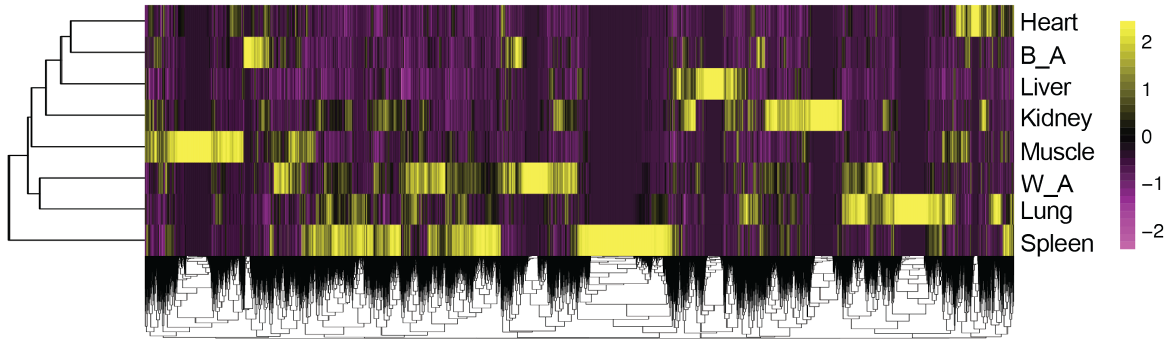

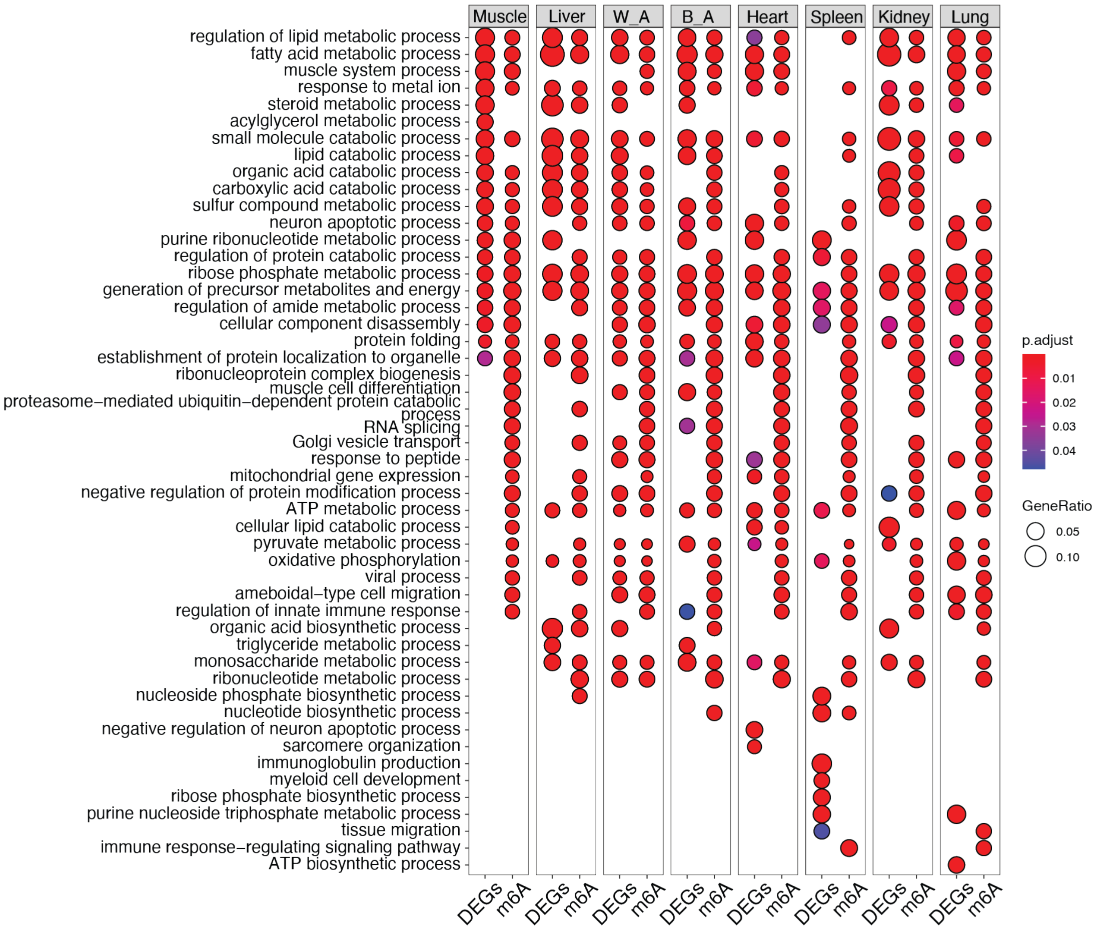

Figure S10 Metabolic responsive dynamics of RNA m6A modification across tissues

(A) Heatmap displaying the frequency of m6A modifications across different tissues, with data normalized by z-score. (B) Comparisons of the top enriched pathways (GO term, Biological Process) for DEGs and transcripts with significantly changed m6A modifications in different tissues during fasting treatments.
